# Physical simulation and theoretical evolution for ground fissures triggered by underground coal mining

**DOI:** 10.1371/journal.pone.0192886

**Published:** 2018-03-07

**Authors:** Jing-Hu Yang, Xiang Yu, Yi Yang, Zeng-Qiang Yang

**Affiliations:** 1 School of Resource and Safety Engineering, China University of Mining and Technology (Beijing), Beijing, China; 2 Beijing Key Laboratory for Precise Mining of Intergrowth Energy and Resources, China University of Mining and Technology (Beijing), Beijing, China; University of Science and Technology Beijing, CHINA

## Abstract

Underground coal mining activities are prone to cause movement and breakage in geological strata and also lead to mining subsidence and even ground fissures. Along the direction working panel advancing, ground fissures may occur in roof in front and/or behind working panel. However, the investigations of previous similarity tests in lab only emphasize on the region behind working panel. By improving strata material property in construction and mounting artificial pressure devices, two physical simulation tests were conducted and successfully investigated the simulated results. Then, the mechanical model of “cantilever beam and elastic foundation beam” was proposed to calculate the stress distribution and the crack initiation angle in overlying strata and it well explains the mechanisms of ground fissures generation and propagation. Results show that, the maximum internal force in roof always occurred in front of working panel. However, because the void space in gob due to excavation is large enough to cause the bend and rotation of roof strata, compare to the triaxially compressed region in front of working panel, the roof always broke off at some positions above gob since the stress concentration resulting from such bend and rotation of strata could easily reach the limit strength of strata rocks. Also, the length of cantilever beam changed dynamically as respect to the panel advancing and the breakage intervals. Thus, the breakage position where the internal force first reached the limit tensile strength is not fixed and there will be two different kinds of relative positions between the crack initiation point and the working panel. The crack initiation direction is always perpendicular to the internal force, and the crack propagation direction is affected by the initiation angle, overburden-separation degree and the position of the hydraulic shields. If there is no overburden-separation or less, the roofs will break off as a composite beam and the propagation direction will be roughly along the central line between the initial broken point and the support position. Otherwise, the roof strata will bend with the support shields moving forward, then the fracture angle will be close to the initiation angle and the fault surface will be stepped.

## Introduction

**Fig 1:**
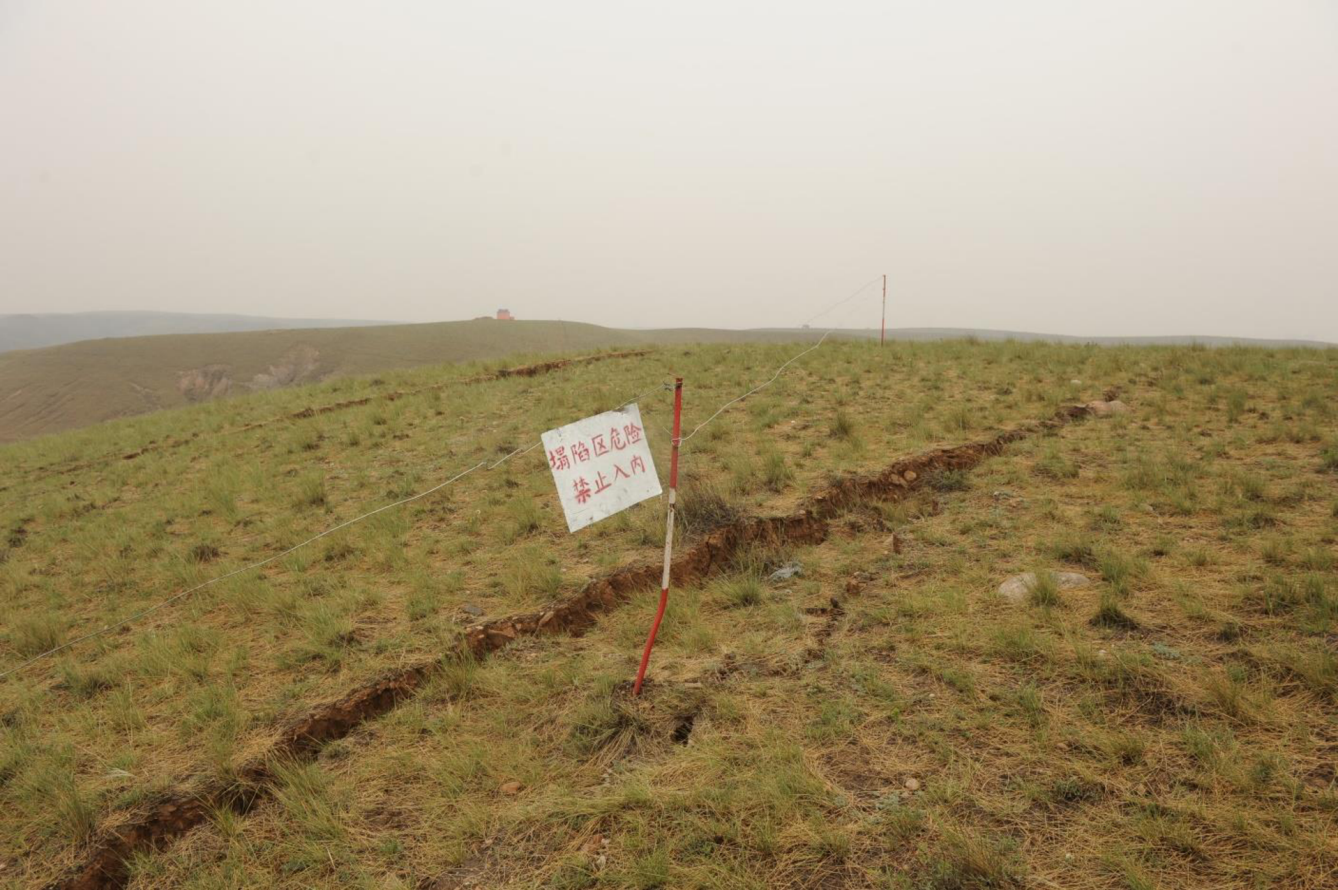
Mining subsidence damages and ground fissures in Wanli Town, Dongsheng District, Erdos City, Inner Mongolia, China.

Underground coal mining activities are prone to cause movement and breakage in geological strata and also lead to mining subsidence and even ground fissures [1–6] (Fig 1). Too much subsidence or ground fissures could cause loss of water resources and destruction of surface vegetation and buildings. And these damages are more serious in longwall mining of shallow coal seam [7–9].

**Fig 2:**
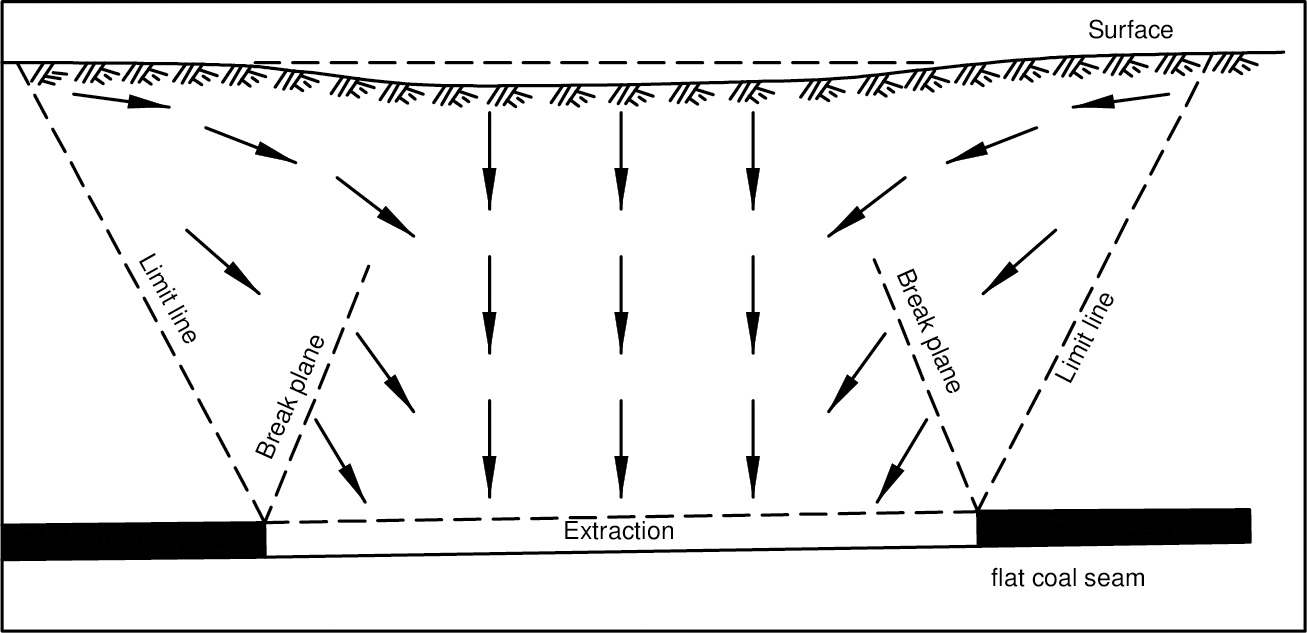
Directions of layer movements caused by subsidence in a flat coal seam.

In order to better predict, measure, and prevent these damages, the mechanism of the formation of ground fissures should be analyzed firstly. The common illustration of the surface subsidence caused by underground mining process is shown as Fig 2 [10]. And the ground fissures will generate and propagate above gob. In fact, there are ground fissures in front of working panels. That is, the ground fissures are not only formed above the break plane, but also above the limit line (shown in Fig 2). For example, in a coal mine in Shuozhou, northern China, according to the measured data on February 21, 2012, a ground fissure was found 30.95m ahead of working panel, and another fissure occurred 52.35m ahead of working panel on March 10 [11, 12].

In order to reproduce and explain these two phenomena, different approaches have been developed, including methods based on influence functions [13–15], profile functions [16], empirical formulas [17], numerical models [18], and geodetic measurements [19, 20]. But research on the physical model and mechanical mechanisms has received less attention and the existing research results are unsatisfactory [21, 22].

**Fig 3:**
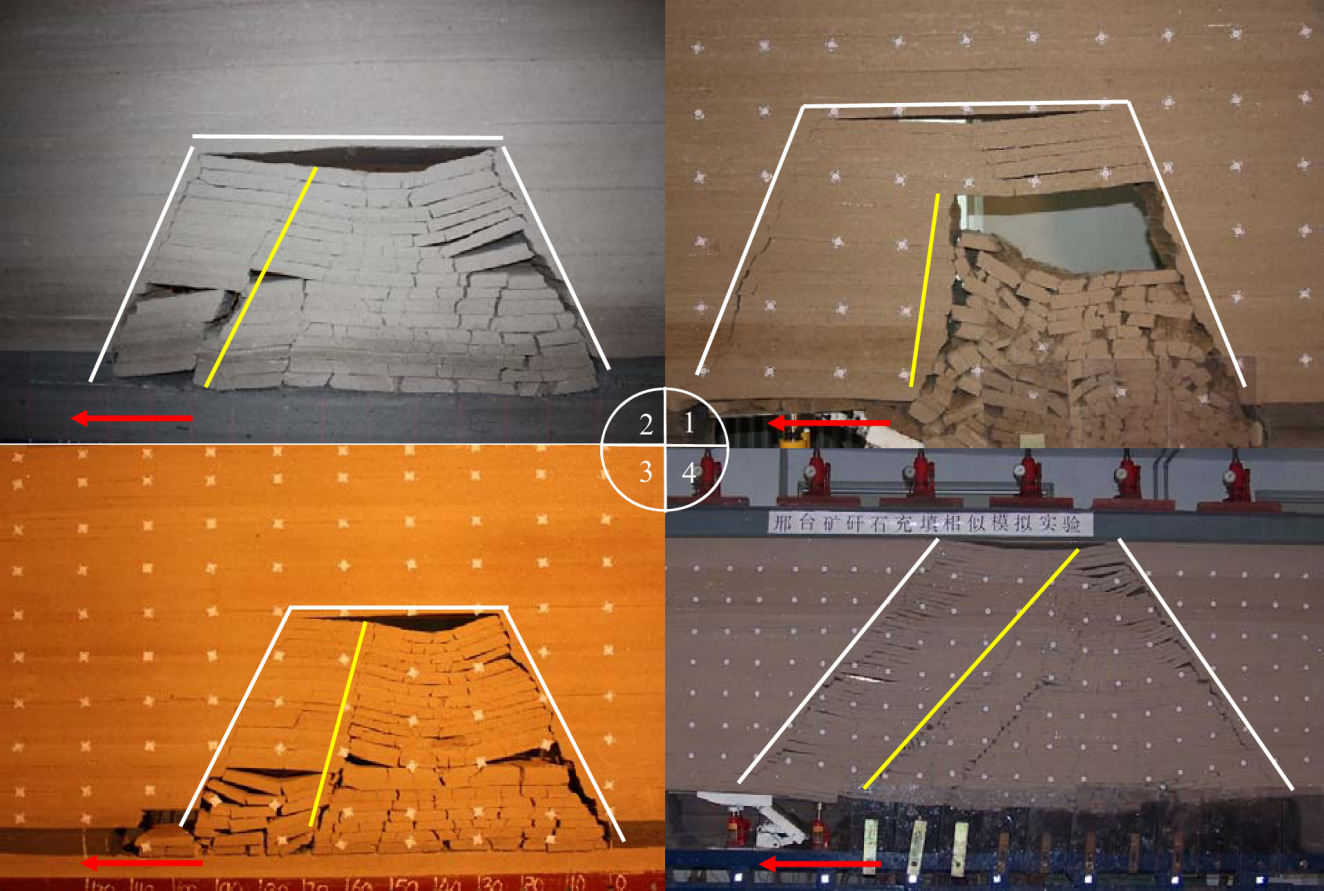
Results of conventional physical similar simulation tests.

Physical similarity simulation is mainly used to build the physical model. It has the characteristics of visible and intuitive results and short experimental period. In order to investigate the fissures, a lot of physical similarity tests were conducted, but for most simulated results, the roof breakage is extended only to gob direction, forming a trapezoidal collapse space, shown as Fig 3. As the bending strata reaches surface, it causes the surface rupture in the direction along the mined-out area. Therefore, the experiment method should be improved to demonstrate more implicated strata breakage.

In this study, two physical simulation tests were conducted and successfully simulated the two kinds of ground fissures after improving simulation material properties and mounting the artificial pressure devices. Then, the characteristics of overlying strata breakage and ground fissures generation were analyzed. Based on the simulated results, the mechanical model of “cantilever beam and elastic foundation beam” was proposed to discuss the mechanisms, and this model can well explain the test results and ground fissures induced in mining process.

## Material and methods

### Improvements of the similarity simulation methods

In conventional similar simulation experiments, the similarity ratio is often larger. While the ratio is 100:1, the size of the model and the pace of the working face to advance in the test should be reduced by 100 times. In underground coal mine, the pace of the working face to advance is mostly 0.8m, then the excavation step in the test is 0.8cm. And the greater the similarity ratio, the smaller the excavation step. In order to facilitate the operation, people often neglected the similarity ratio of the excavation step. As a result, the step is too large for the model size and material strength, that's where the mistake is. Assuming that the excavation step is maintained for this large setting, the strength of the material should be increased accordingly.

Thus, we made the following improvements.

1. The mixture ratio of similarity materials is improved by using suitable amount of cement to increase the model strength.
2. Artificial pressure will be used to offset the error caused by increased strength.

http://dx.doi.org/10.17504/protocols.io.jwycpfw. [PROTOCOL DOI]

### Material preparation and model building

In order to increase the credibility of the experimental results, two different tests were conducted to get the similar results.

#### Test 1

Considering the maneuverability, the geological conditions of coal seam as shown in Table 1 were designed.

**Table 1:** Geological conditions in model (ratio of similitude 100:1).

Layer No.	Lithology	Real thickness(m)	Simulation thickness(cm)	Simulation materials number
10	Mudstone	10.0	10.0	A
9	Packsand	13.0	13.0	B
8	Mudstone	4.0	4.0	A
7	Medium sandstone	13.0	13.0	C
6	Mudstone	8.0	8.0	A
5	Packsand	13.0	13.0	B
4	Mudstone	8.0	8.0	A
3	Coal	4.0	4.0	D
2	Packsand	2.0	2.0	B1
1	Packsand	6.0	6.0	B1
Total thickness		81.0	81.0	

According to the simulation theory, analog simulation materials mainly consist of aggregate and cementing materials. Aggregate is a big part of similarity material. Its physical and mechanical properties have important effects on the density of similarity materials. According to the field geological data of coal mine and simulation theory, fine sand was used as aggregate in this study. Cementing materials are the dominant elements affecting material’s strength, and we used gypsum, lime and cement as cementing materials.

**Fig 4:**
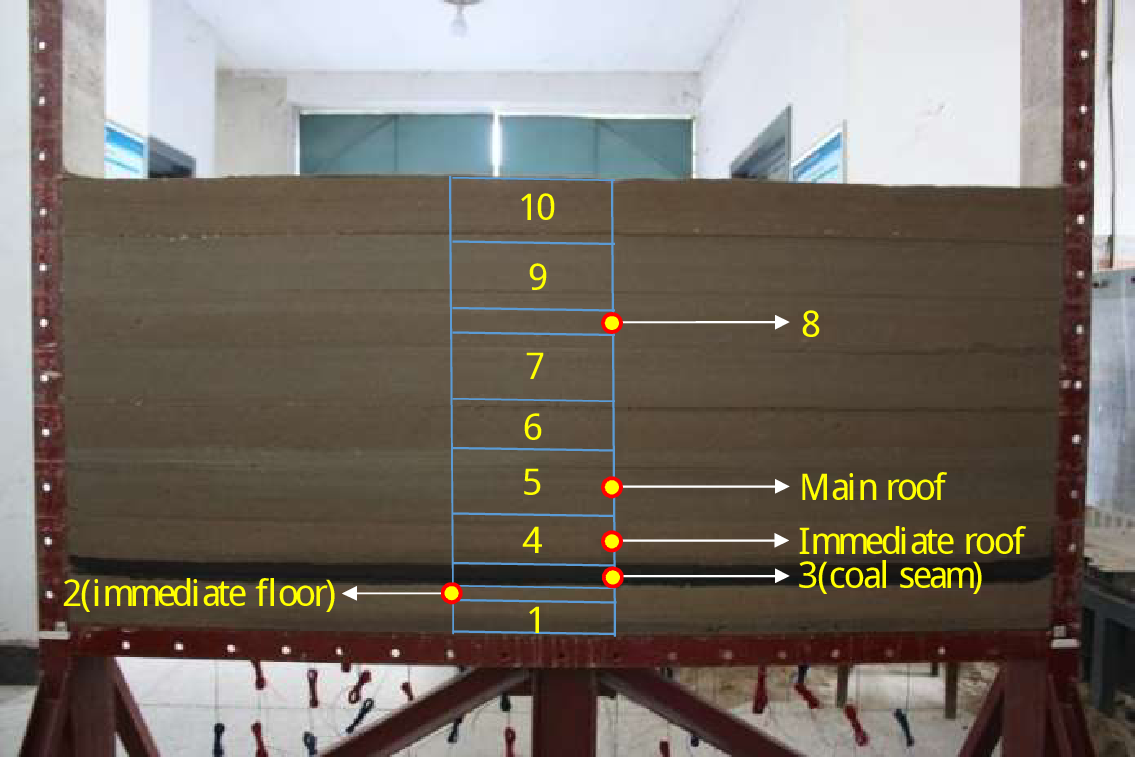
The accomplished two-dimensional plane model.

As shown in Fig 4 and Table 2, after a lot material mixture experiments, we found the proper material mixture ratio finally.

**Table 2:** Similar material properties.

Simulation materials No.	The uniaxial compressive strength(MPa)	Tensile strength (MPa)	Material mixture ratio (volume-weight ratio)				
			Sand	Gypsum	Lime	Cement	Water
A	1.14	0.22	40	3	2	2	4.7
B	1.59	0.32	12	1	1	1	1.5
C	1.21	0.31	14	1	1	1	1.7
D	0.62	0.19	80	7	3	0	6.3
B1	0.80	0.19	12	1	1	0	0.98

According to the geological conditions of coal seam and the proper material mixture ratio, we made proper similarity materials and compacted them into the final mode, which dimension is 1800mm×160mm×810mm, as shown in Fig 4.

**Fig 5:**
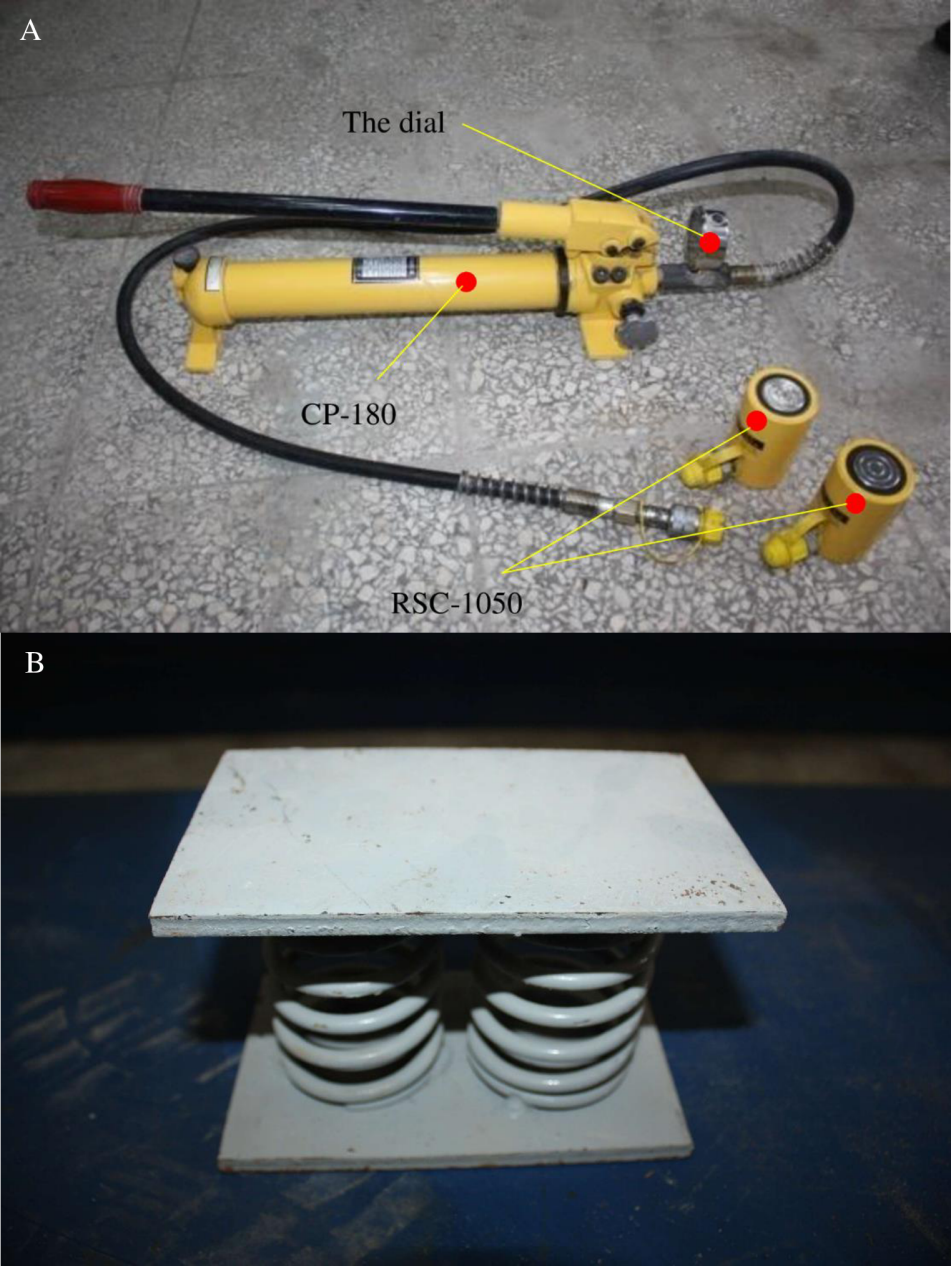
The hydraulic loading devices. (A) “RSC–1050” jacks and “CP–180” hydraulic pump. (B) Spring-steel plate mechanisms.

And in Test 1, four “RSC–1050” jacks and one “CP–180” portable ultrahigh pressure hydraulic pump were used to load on the model. And a few spring-steel plates were used to realize loading continuity. They are shown in Fig 5 and Table 3.

**Table 3:** “RSC–1050” jacks’ parameters.

Type	RSC–1050
**Output tonnage**	10t
**Active area**	15.9cm^2^
**Oil cylinder inner diameter**	45mm
**External diameter**	63mm
**Weight**	2.4kg
**Height**	106mm
**MAX. stroke**	50 mm

The “CP–180” portable ultrahigh pressure hydraulic pump is equipped with four hydraulic splitters and one dial, and could load same pressure on four jacks at the same time. The spring-steel plate mechanisms could change work done by jacks into strain energy storing in the springs and realize the continuous load on model with the mining subsidence. The springs’ height is 110 mm and carrying capacity is 150kg full scale, which fully meets the requirements of the test.

**Fig 6:**
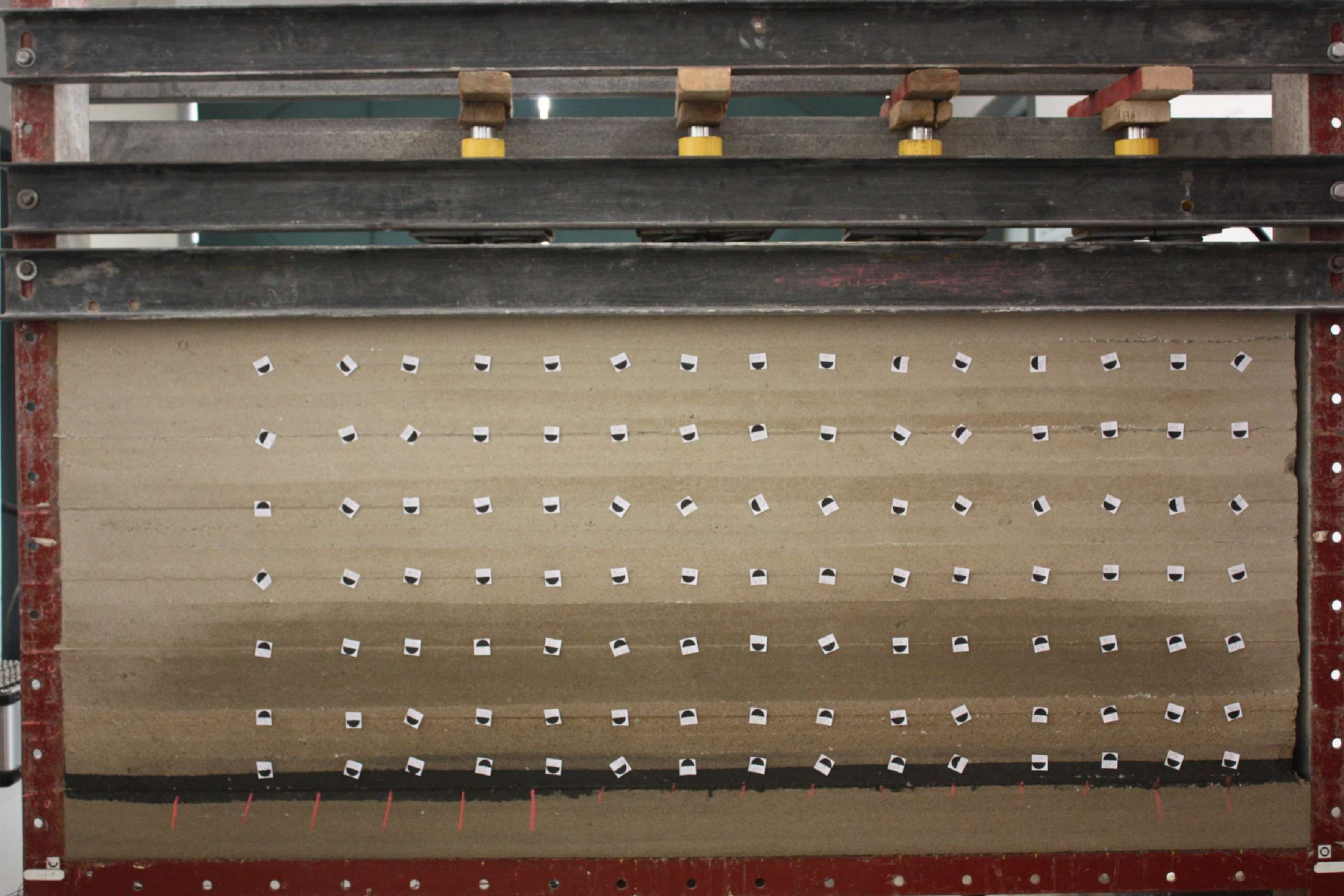
The final model of Test 1.

The final model is shown as Fig 6. And we plan to excavate 140 mm in coal seam from right to left, and observe roof movement and breakage during excavation.

#### Test 2

In order to make the simulation experiment more close to field situation, the loading system has been improved, and the simulation part of the hydraulic support has been used.

**Fig 7:**
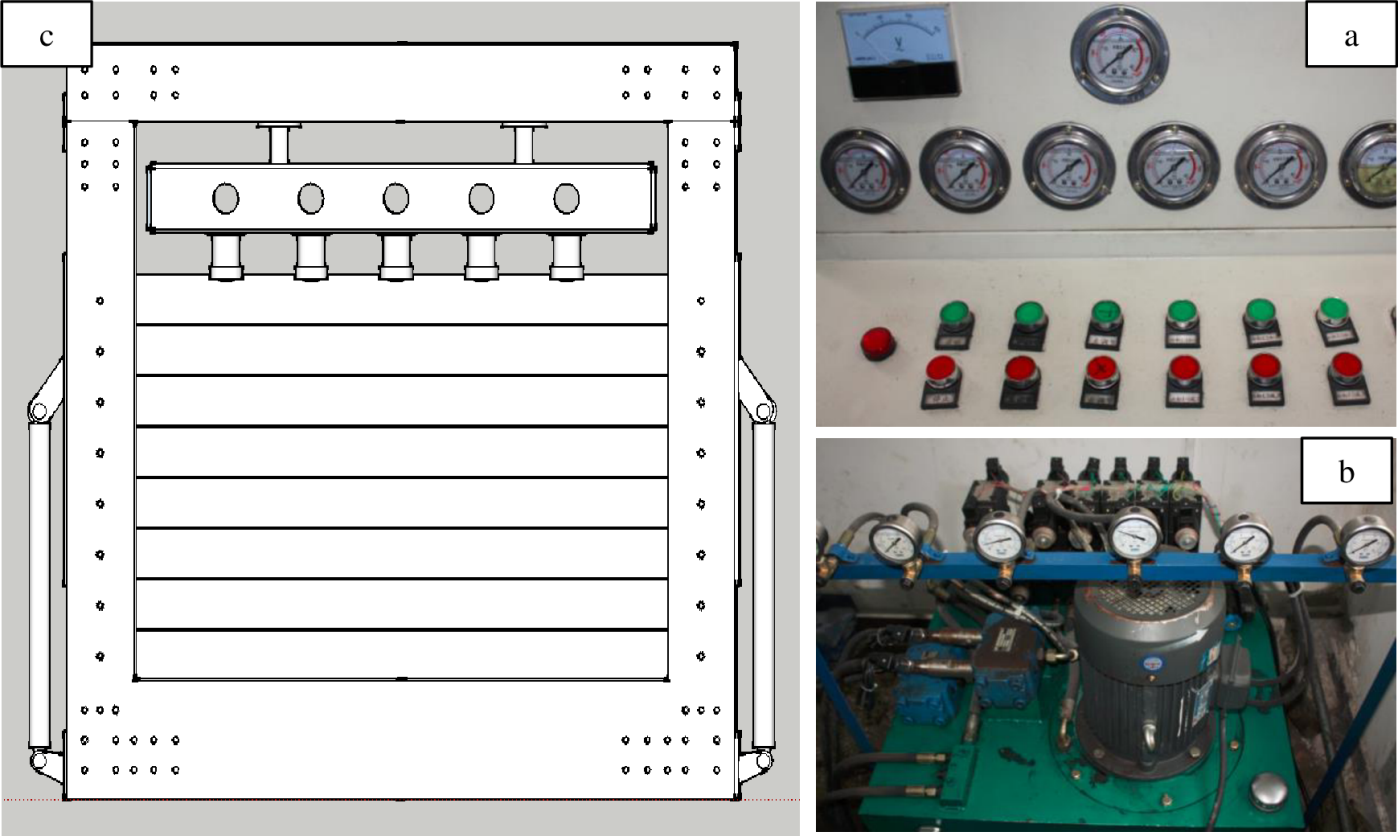
Quasi-three-dimensional high-pressure hydraulic loading simulation experiment platform.

As shown in Fig 7, the maximum size of the model made via this platform can be 2470mm×1800mm×40mm, which is twice the width of the traditional two dimensional simulation platform and can effectively reduce the boundary effect of the model. At the top of the platform, an adjustable hydraulic loading system is set up, which can be applied to simulate the variation pressures under different conditions.

**Fig 8:**
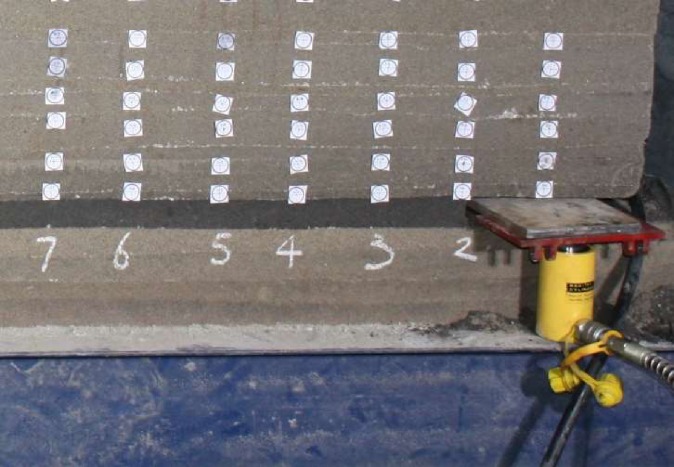
The simulation hydraulic support with working resistance monitoring sensor.

The simulation hydraulic support is composed of two “RSC–1050” jacks and one “CP–180” portable ultrahigh pressure hydraulic pump, and a working resistance monitoring sensor is set under the canopy of each support (Fig 8). The two supports are connected with a steel plate to support the coal body.

And in this test, a plane strain model with geometric similarity ratio α_*L*_ = 100:1 is used to simulate the movement and breakage of overlying strata (Tables 4 and 5, S1 and S2 Files)

**Table 4:** Geological conditions in model (ratio of similitude 100:1).

Layer No.	Lithology	Real thickness(m)	Simulation thickness(cm)	Simulation materials number
12	Aeolian sand	15.25	15.25	F
11	Loess	35.5	35.5	G
10	Coarse sandstone	31.2	31.2	H
9	Mudstone	2	2	A
8	Siltstone	5.12	5.12	I
7	Mudstone	1.1	1.1	A
6	Fine sandstone	2.9	2.9	B
5	Medium sandstone	3.9	3.9	C
4	Siltstone	5.45	5.45	I
3	Mudstone	0.5	0.5	A
2	Coal	3.2	3.2	D
1	Mudstone	12	12	A1
Total thickness		118.12	118.12	

**Table 5:** Similar material properties.

Simulation materials No.	The uniaxial compressive strength(MPa)	Tensile strength (MPa)	Material mixture ratio (volume-weight ratio)				
			Sand	Gypsum	Lime	Cement	Water
F	\	\	1	0	0	0	0
G	0.54	0.17	90	5	5	2	10.2
H	0.89	0.34	70	4	7	5	8.6
I	1.38	0.29	60	7	5	5	7.7
A1	0.80	0.28	40	3	2	0	3.15

**Fig 9:**
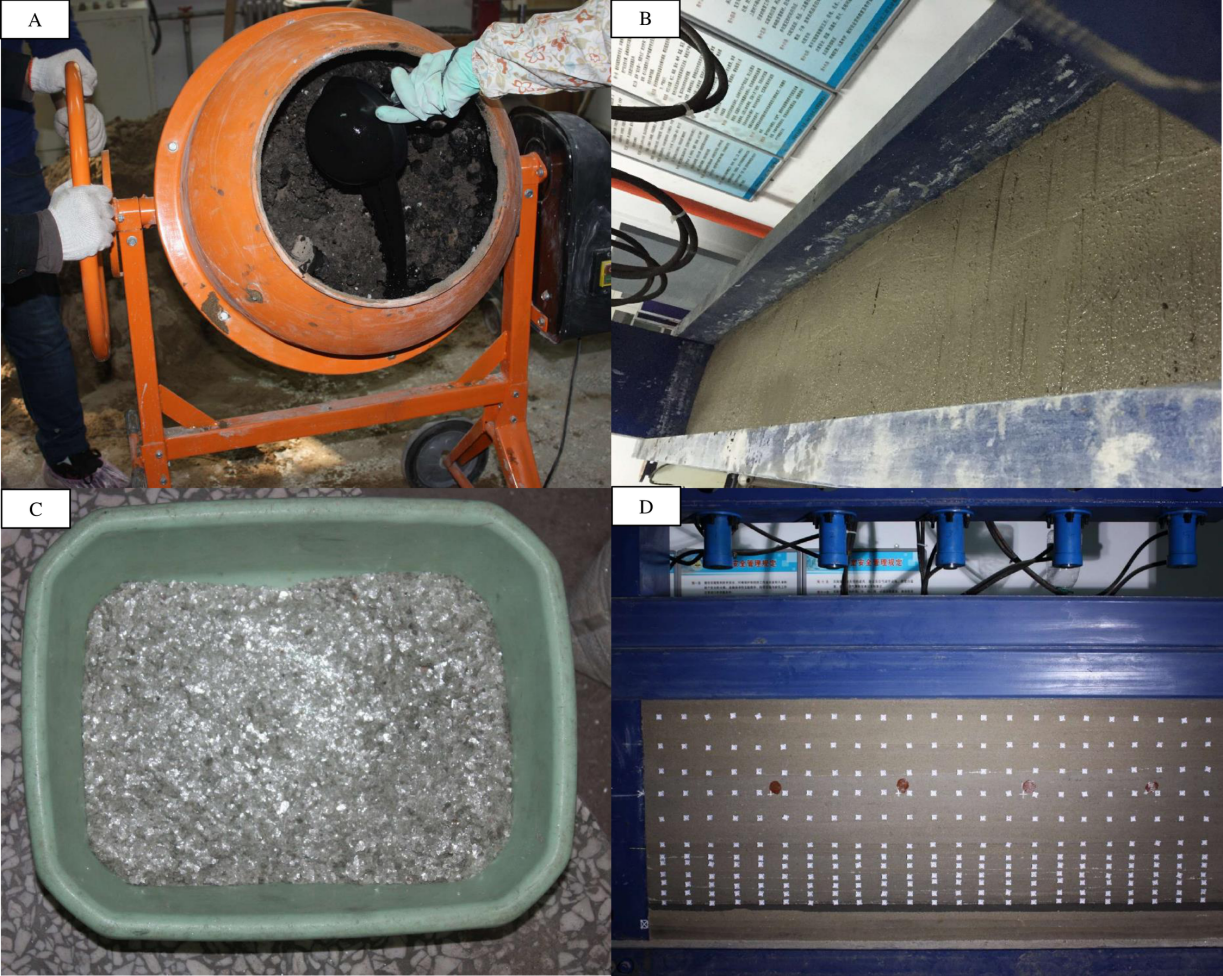
The physical model of Test 2. (A) Mixture of materials. (B) Compaction of materials. (C) Mica powder for layer separation. (D) The accomplished model.

The physical model building process is shown as Fig 9. Before excavation, a uniform load of 0.008MPa is applied to the surface by the electro-hydraulic loading system, and the excavation step is 10cm.

### Model boundary processing

**Fig 10:**
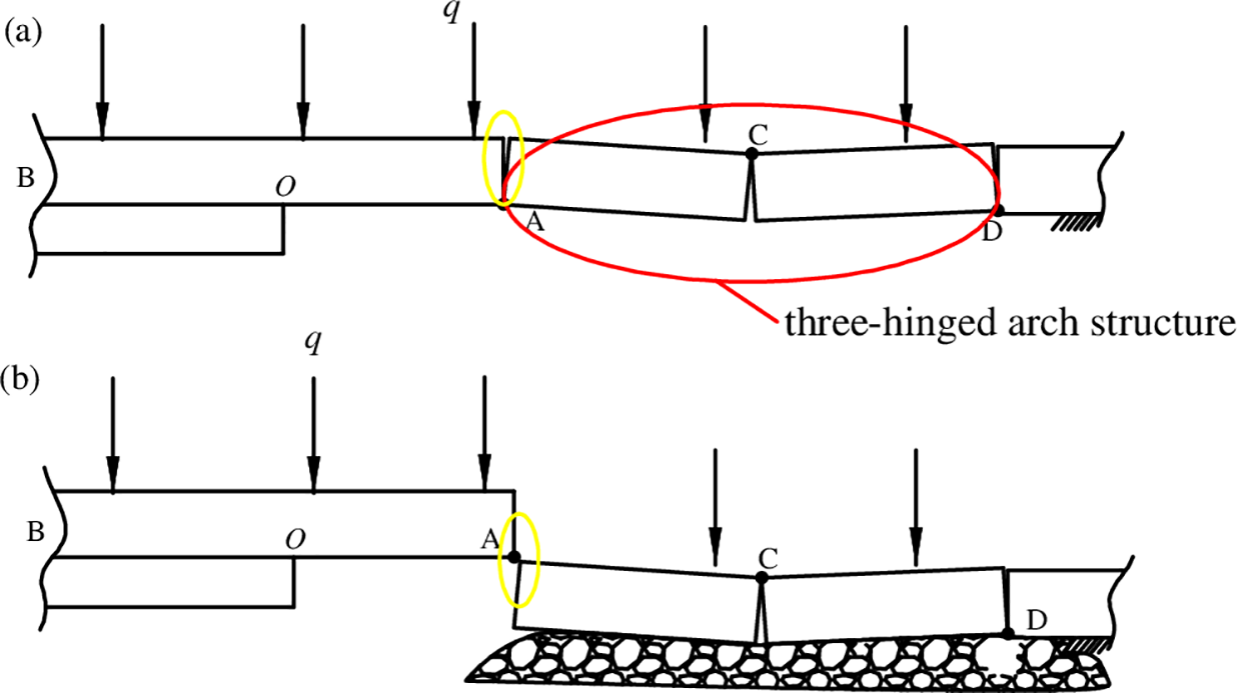
Two cases with and without three-hinged arch structure.

**Fig 11:**
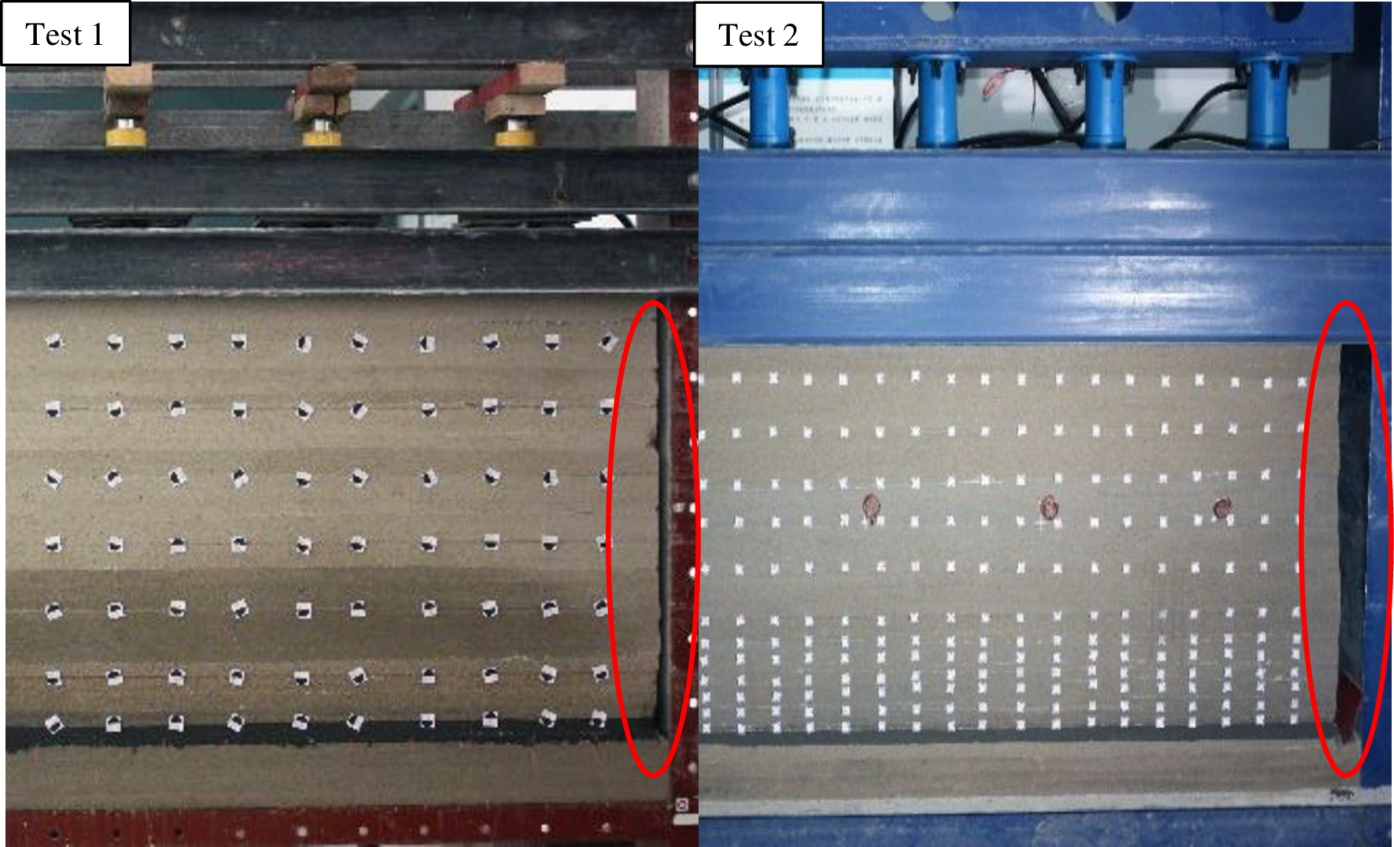
Slots set on the right side of the models.

The coal measure strata are formed via sedimentation and diagenesis, whose lithology, thickness and strength are different from one stratum to another. A series of strata behavior such as deformation, breakage, overburden-separation, surface subsidence and ground fissures are mainly controlled by adjacent strata. Underground coal mining excavates coal seam and creates void spaces, making the overlying strata losing support and breaking and then may forming a mechanical structure of “three-hinged arch”, as shown in Fig 10. Due to the structure, the right end A of the unbroken strata will be acted upon by forces. If the above structure cannot be formed, or the hinge point cannot withstand the overburden load, the broken rock block will fall to the mined area. In this case, since the contact between the rock blocks above the hinge point A is weak before the broken rock block fell, the contact force between the rock block OA and the fallen rock block AC is small even could be ignored. After the roof OA broken, the rock block OA will contact with the fallen block AC at the right end and be acted upon by forces again. In order to simulate these two cases at the same time, a small slot will be set up on the right side of the models as shown in Fig 11. Then, the roof’s first breaking can simulate the case without three-hinged arch structure, and others can simulate the case with three-hinged arch structure.

## Results and discussion

### Test 1

**Fig 12:**
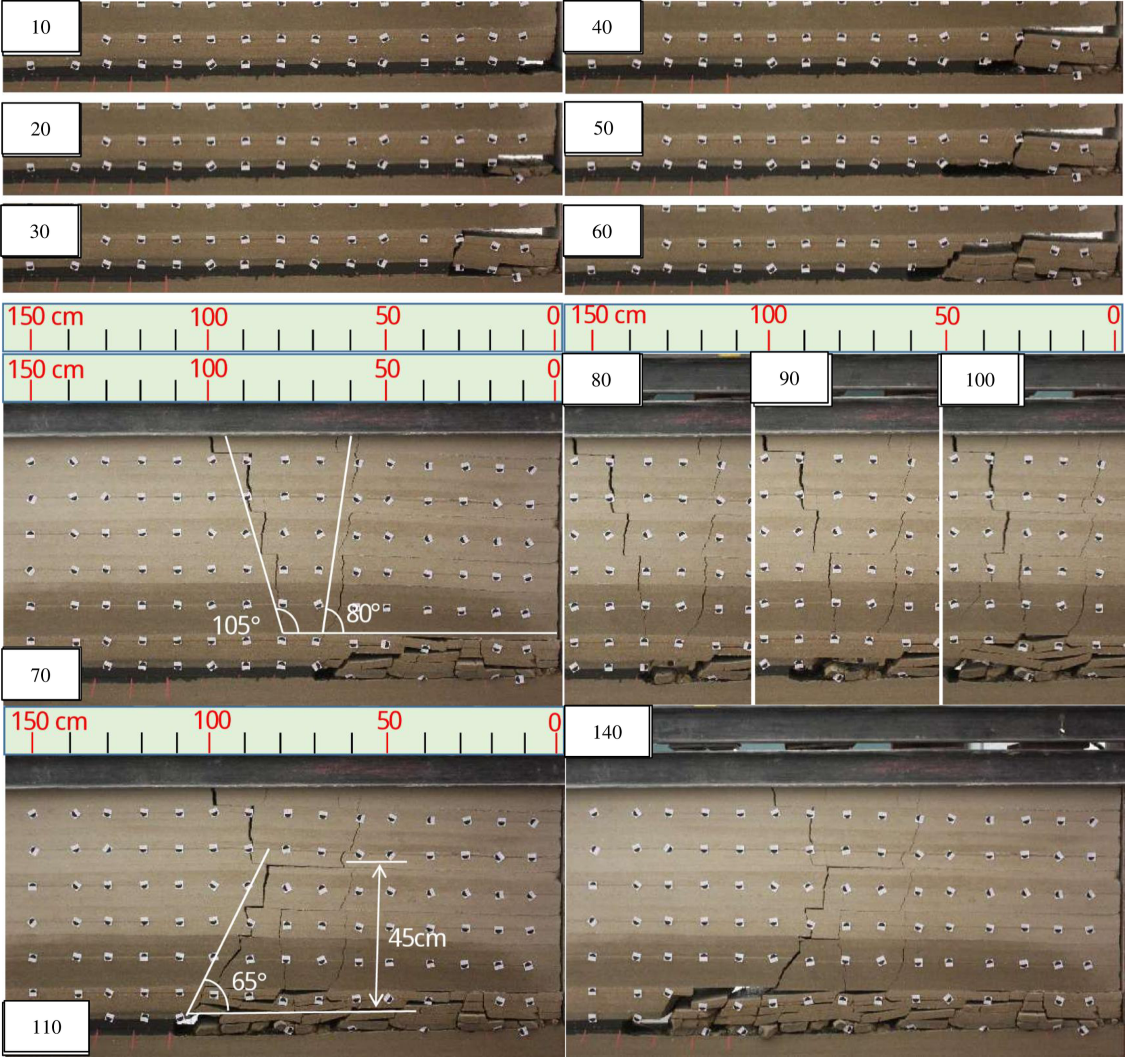
Overlying strata movement and breakage during the excavation.

During the excavation (Fig 12), the immediate roof falls instantaneously, forming a small trapezoidal collapse space. When excavated to 70cm, the overlying strata broke at a wide range with a severe sound forming fault lines through the surface Two opposite direction fault lines almost generated at same time. One is to the working panel advancing direction, and the other is to the direction of the mined-out area. The angle between the fault line and the horizontal direction is about 80° and 105°, respectively.

As the working panel advanced, the overlying strata in the mining area rotated and sank, and gradually compacted the falling rocks in gob. When excavated to 100cm, a new fault line occurred and propagated obviously at 110cm. The angle between the new fault line and the horizontal direction is about 65°, and the height of the new trapezoidal collapse space is 45cm. Keeping advancing, the roof produced a more substantial rotation, resulting in the cracks of overlying strata gradually squeezed.

**Fig 13:**
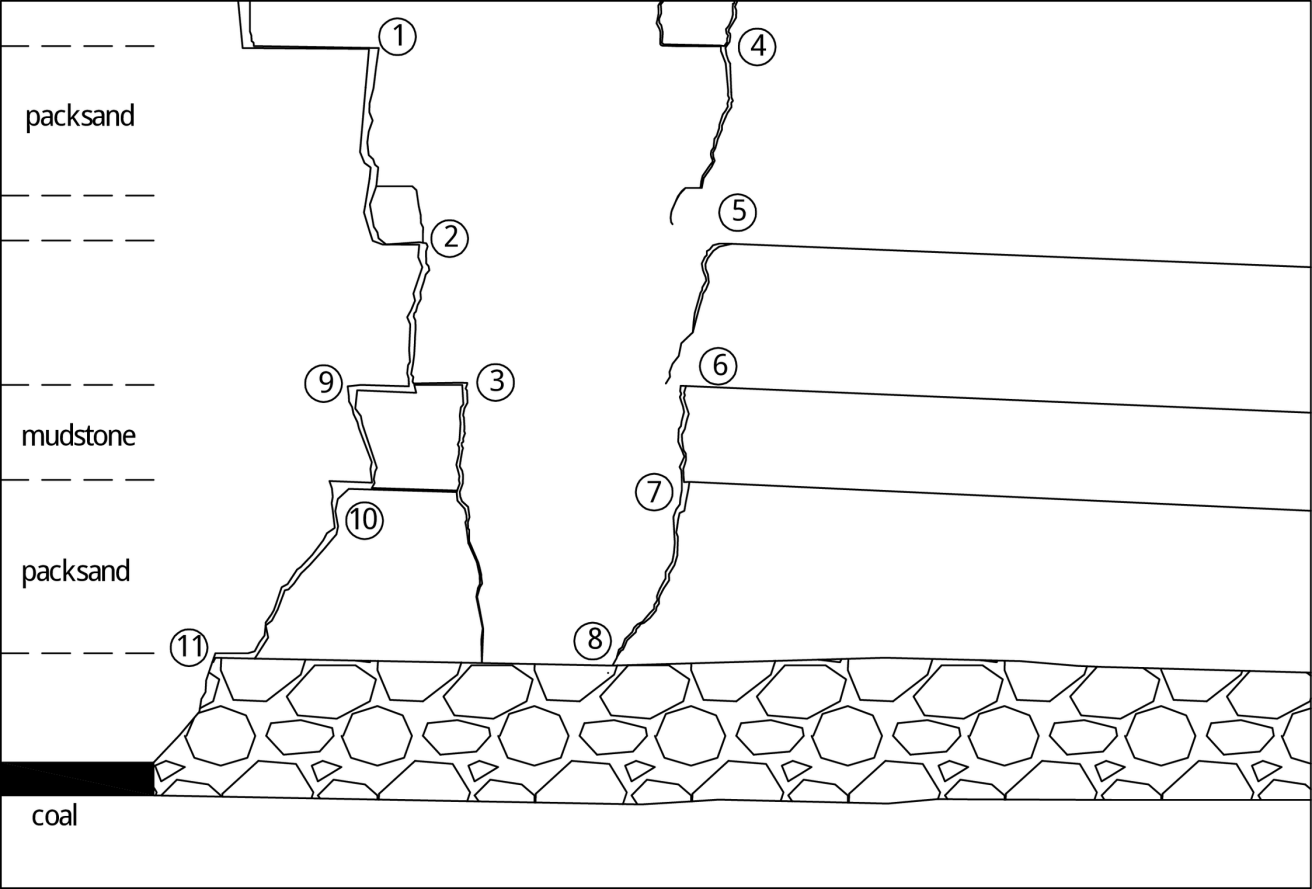
Overlying strata movement and breakage during the excavation.

In the test, it can be seen that the fault line is stepped, indicating that the breakage of the overlying strata is accompanied by overburden-separation, which causes the upper layer to break ahead of the lower break position. And each layer breaks from its top with different crack initiation angles. As shown in Fig 13 and Table 6, the distance of fracture ahead and the initiation angle varies respect to the position. The layer begins to break at initiation angle, and then cracks extend to the lower support point, so the final fracture angle may not be the same as the initiation angle. The final fracture direction may be forward or backward or almost vertical.

**Table 6:** The distance of fracture ahead, initiation angle and fracture angle at different position.

Sequence No.	Distance of fracture ahead(mm)	Initiation angle(°)	Fracture angle(°)	Sequence No.	Distance of fracture ahead(mm)	Initiation angle(°)	Fracture angle(°)
1	10.6	83	91	6	1.5	90	92
2	3.8	78	85	7	0.0	89	70
3	4.8	85	85	9	-4.9	86	102
4	5.1	69	72	10	-3.1	88	60
5	3.4	33	69	11	-3.4	72	64

Note: “-”means the opposite direction.

### Test 2

**Fig 14:**
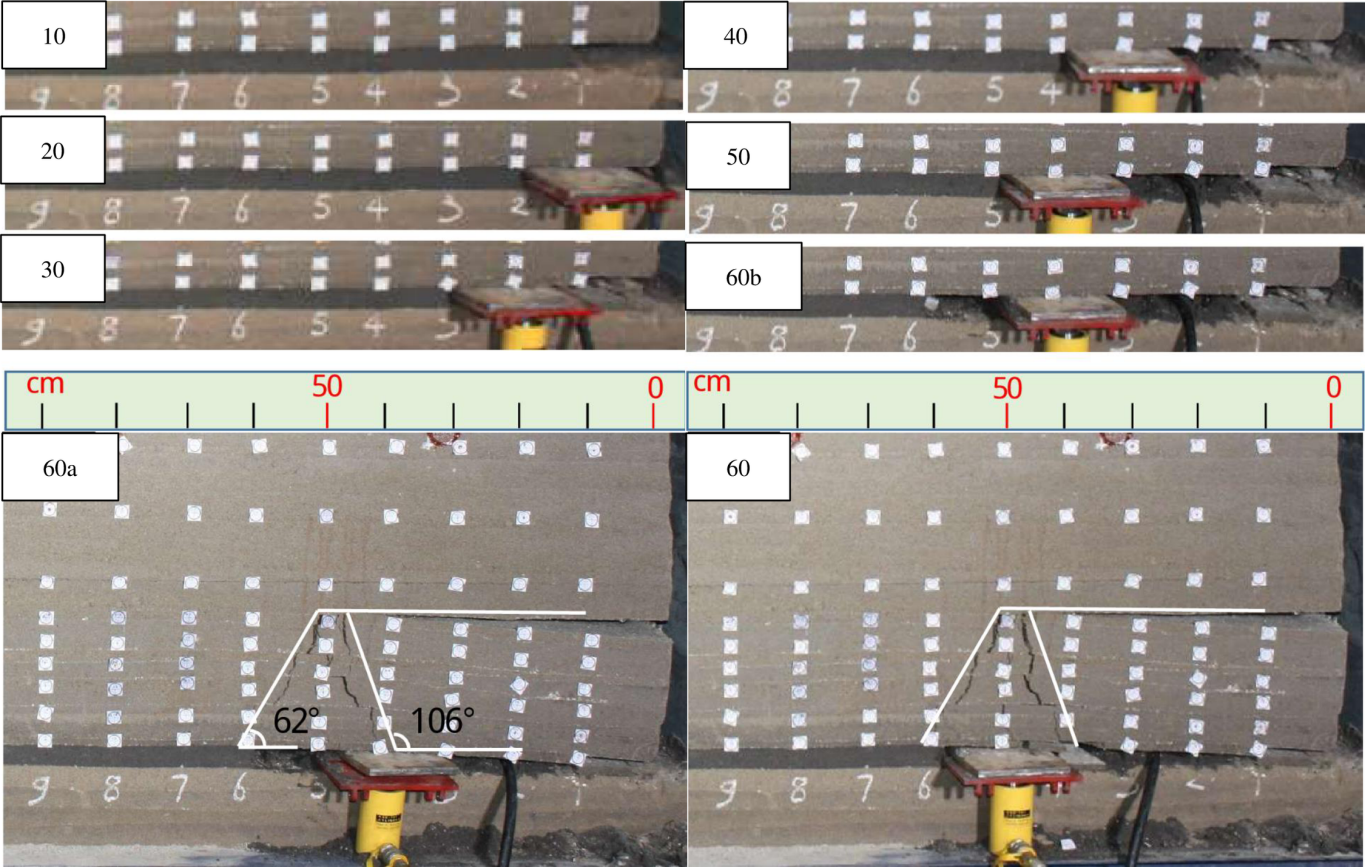
Overlying strata movement and breakage before excavated to 70cm. Note: “60b”means the coal wall is at 60cm but the support is at 50cm and doesn’t lower, “60a”means the coal wall is at 60cm and the support lowers at 50cm.

As shown in Fig 14, there were only immediate roofs falling from time to time before excavating to 60cm, and the main roofs had no obvious bending. When excavated to 60cm, the main roofs under 10^th^ strata suddenly broke while the support was lowering. And two different direction fault lines were formed in the breaking position almost at the same time. The angle between the fault line and the horizontal direction is about 62° and 106°, respectively.

**Fig 15:**
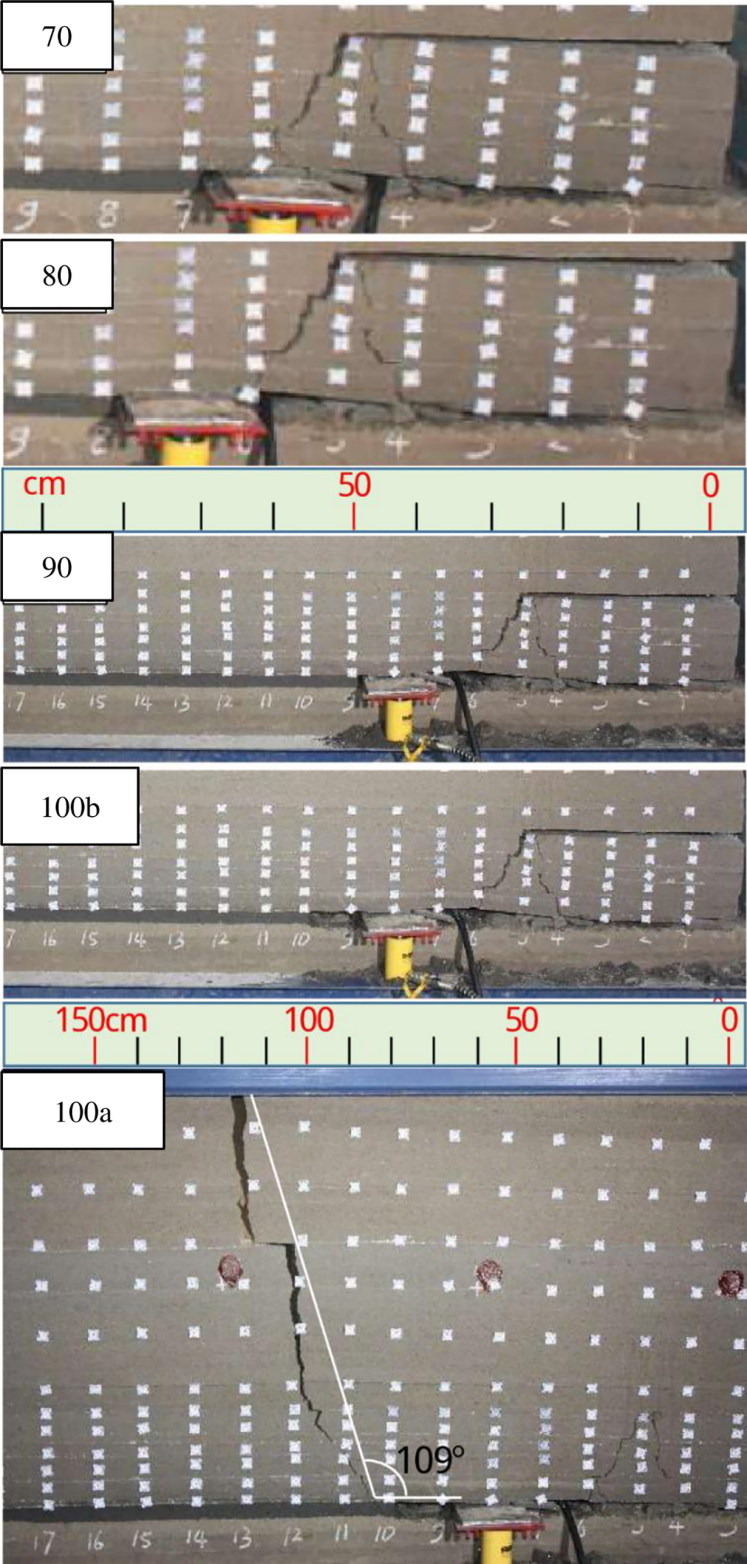
Overlying strata movement and breakage when excavated to 70–100cm.

When excavated to 100cm after the support lowered (Fig 15), the overlying strata broke at a wide range with a severe sound and a fault line to the surface, reaching to the fully mining state. And the long fault line was to the mining working face advancing direction, whose angle with the horizontal direction is about 109°.

**Fig 16:**
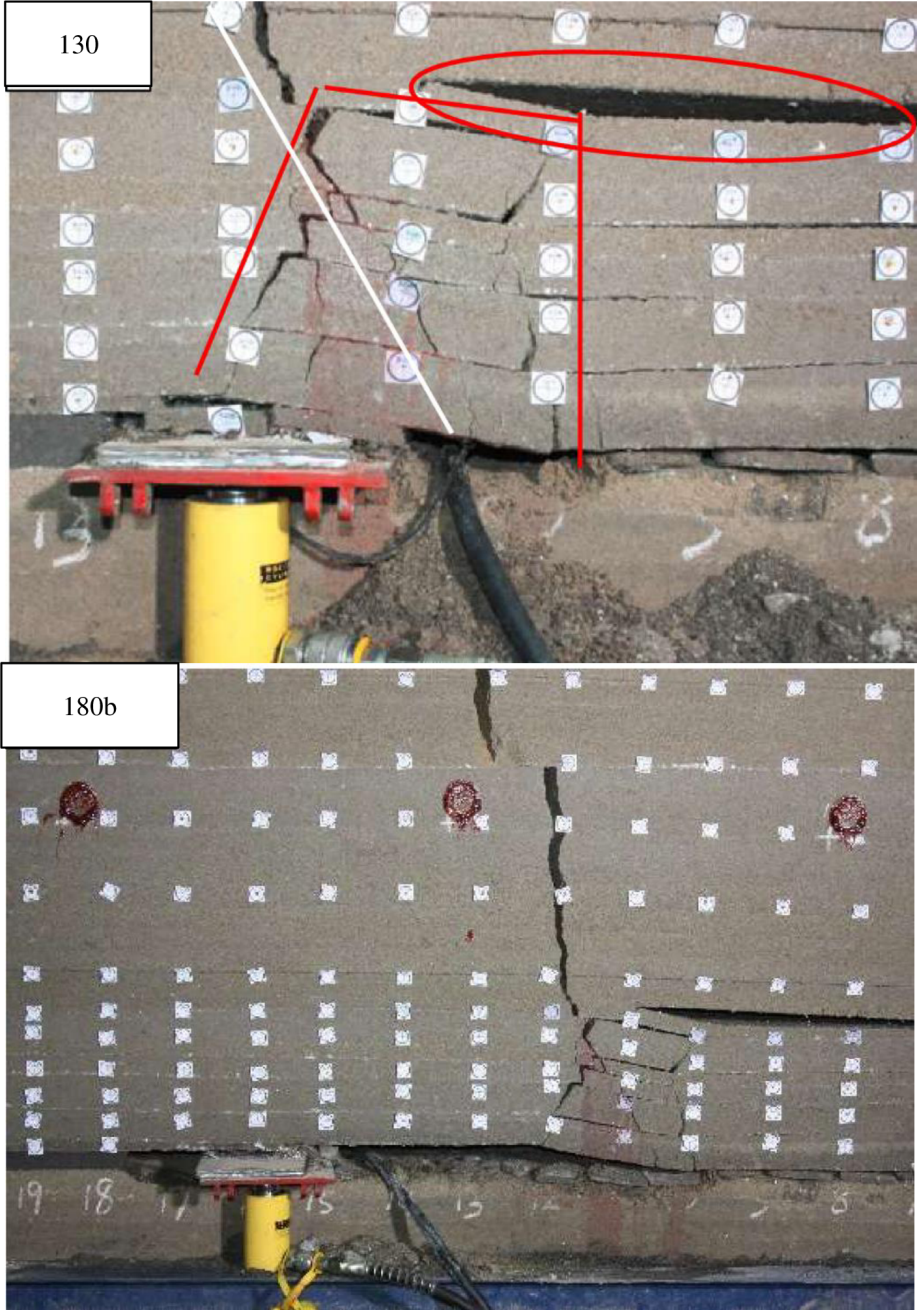
Overlying strata movement and breakage when excavated to 130cm and 180cm.

When excavated to 130cm (Fig 16), the roofs under 10^th^ strata broke with overburden-separation, forming a small trapezoidal collapse space.

**Fig 17:**
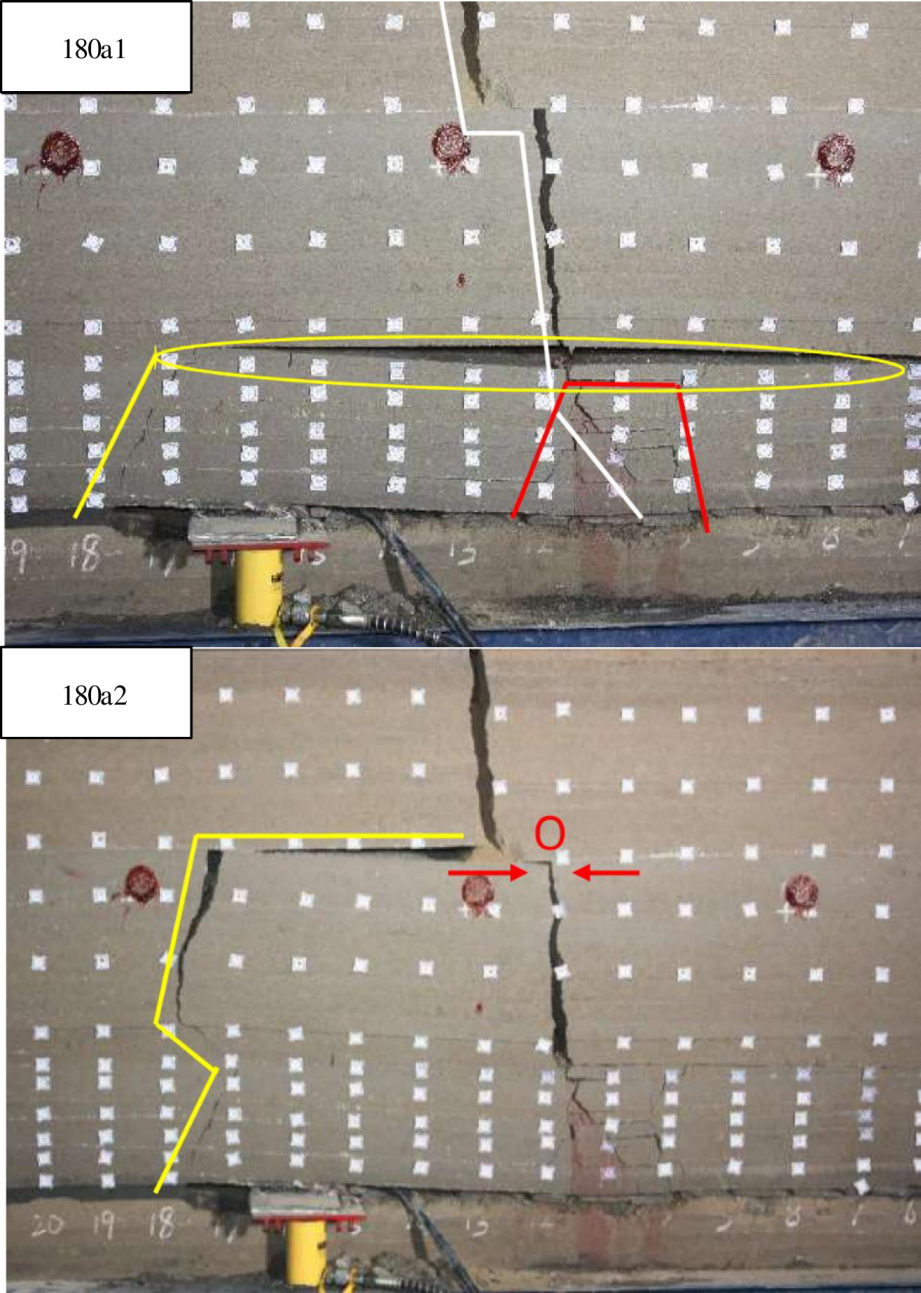
Overlying strata movement and breakage when excavated to 180cm.

When excavated to 180cm and the support started to lower (Fig 17), the roofs under 10^th^ strata broke with overburden-separation and a fault line to the direction of the mined-out area. Then the 10^th^ strata bended and broke as the support continued to lower. And the overlying strata kept stable until broken rocks squeezed and closed the space caused by overburden-separation.

**Fig 18:**
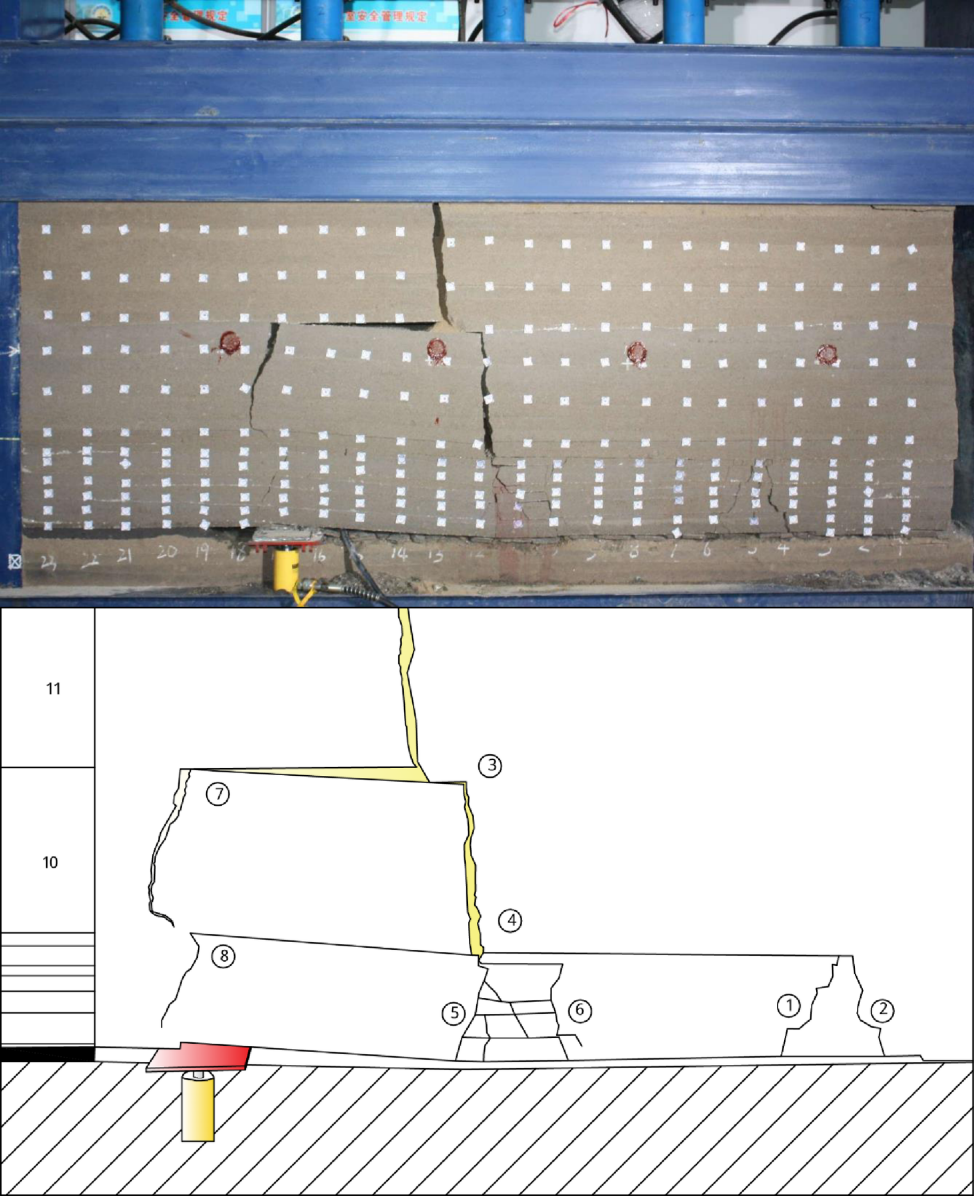
Overlying strata movement and breakage after finished excavation.

The final breakage formation of overlying strata after finished excavating is shown as Fig 18. And the distance of fracture ahead, initiation angle and fracture angle in this test is shown in Table 7.

**Table 7:** The distance of fracture ahead, initiation angle and fracture angle at different position.

Sequence No.	Distance of fracture ahead(mm)	Initiation angle(°)	Fracture angle(°)	Sequence No.	Distance of fracture ahead(mm)	Initiation angle(°)	Fracture angle(°)
1	-2.0	74	60	5	-1.8	66	69
2	2.0	72	108	6	0.0	82	101
3	7.7	90	92	7	\	90	79
4	1.2	86	121	8	4.1	66	64

Note: “-”means the opposite direction.

**Fig 19:**
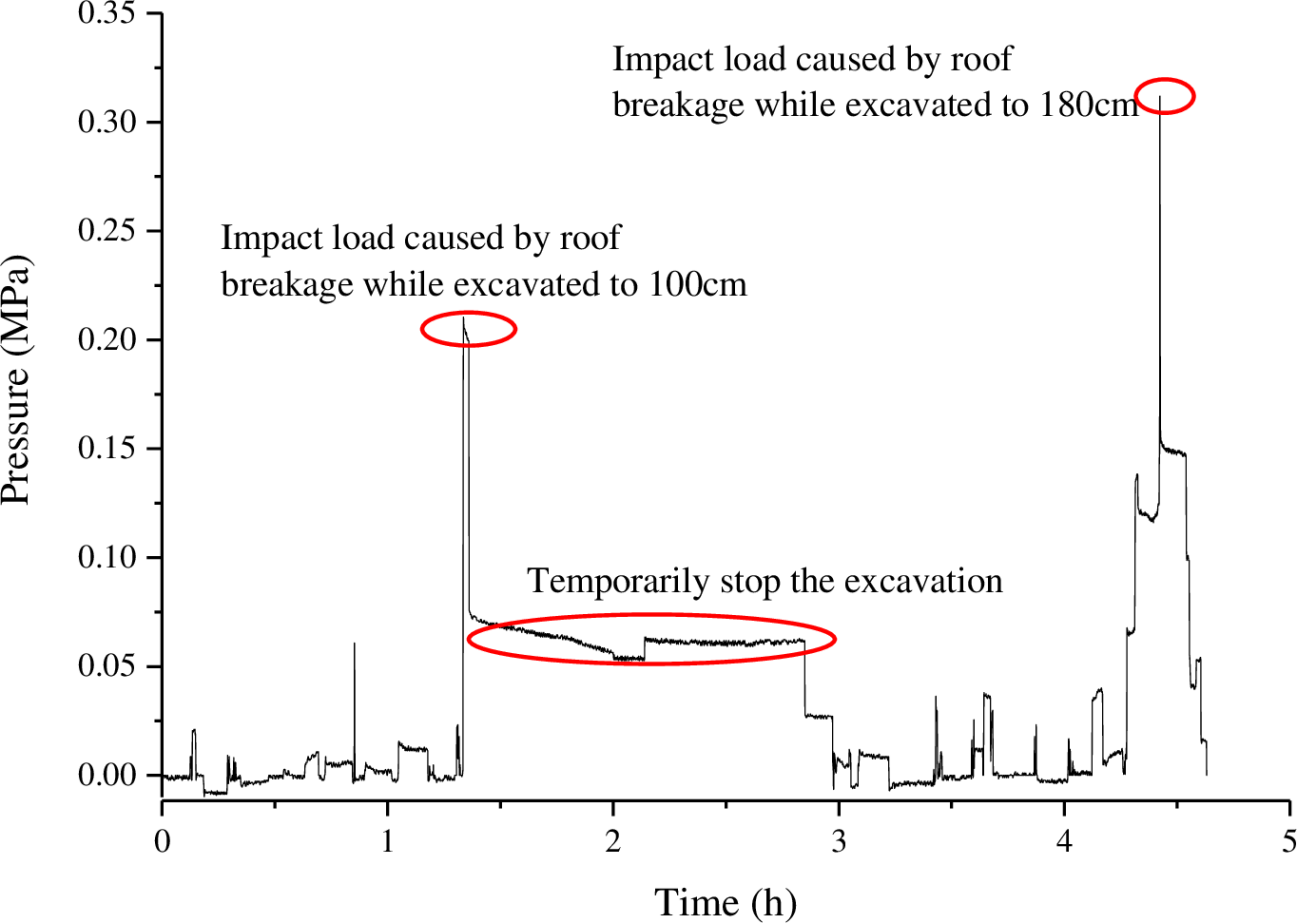
The stress of the hydraulic support during the caving process.

The stress of the hydraulic support during the caving process is shown as Fig 19 (S3 File).

### Characteristics of overlying strata’s movement and breakage

According to the above test results, it can be found that there are the following characteristics in the movement and breakage of overlying strata.

1. When the working face advances to a certain distance, the roof breaks;
2. The breakage and collapse space varies with the different mining state, some of which is small, and some of which directly affects the surface;
3. Overlying strata breakage mostly occurs when the support lowering;
4. The relative position of the crack initiation point and the coal wall is not fixed. Some crack initiation point is generated in front of the coal wall, and some is back.
5. The crack initiation angle varies with the different position, and affects the final fracture angle. But the final fracture angle is mostly different from the initiation angle, and some of which is acute angle, and some is obtuse angle, and sometimes one acute angle and one obtuse angle at the same time.
6. The roof breakage is often accompanied by overburden-separation and the phenomenon of fracture ahead. And the greater the degree of overburden-separation, the big the distance of fracture ahead. If the degree is small or even no overburden-separation, the roofs will break as a composite roof.

### Theoretical analysis

#### Mechanical model

**Fig 20:**
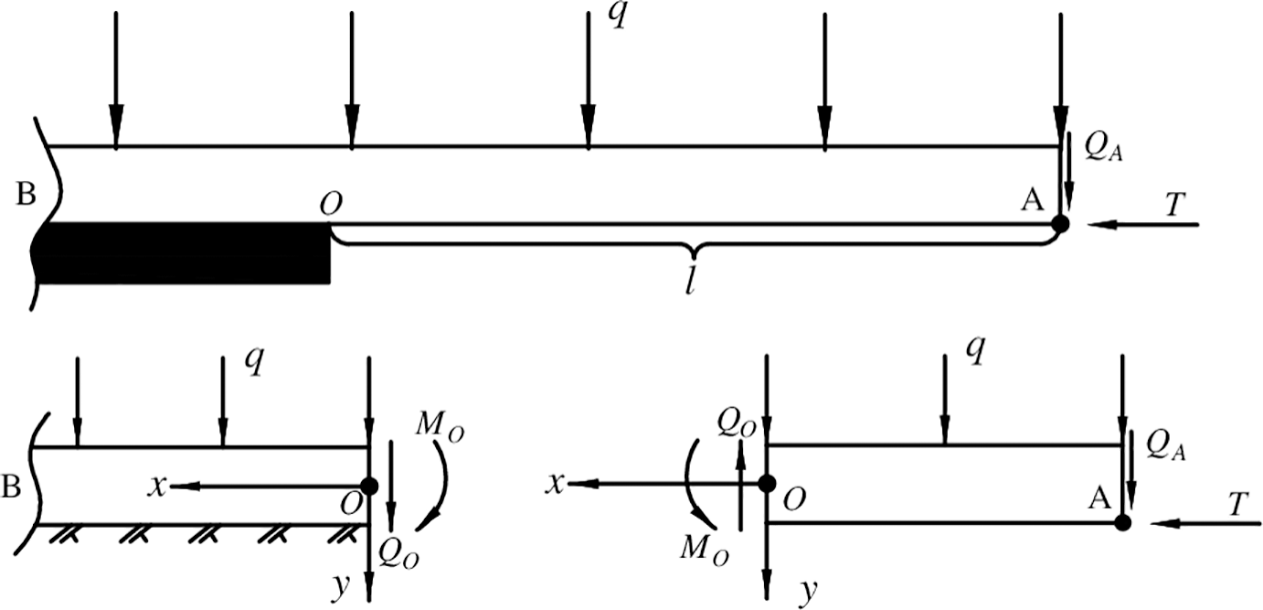
Mechanical model of roof’s fracture.

The two cases in Section “Model boundary processing” can be both explained by a mechanical structure of “cantilever beam and elastic foundation beam”. As shown in Fig 20, the OA segment is a cantilever beam structure. The OB segment is a semi-infinite elastic foundation beam structure with uniformly distributed load. The thickness of the roof is *h*, the length of OA is *l*, the elastic modulus is *E*, the section moment of inertia is *I*, the tensile strength is *σ*_t_, and the uniformly distributed load is *q*. The deflection, rotation angle, bending moment and shear force at O in the beam are *y*_*0*_, *θ*_*0*_, *M*_0_ and *Q*_0_, respectively. The horizontal and vertical components of the force at A in the beam are *T* and *Q*_A_, in the case without three-hinged arch structure, they are all zero. The positive directions of bending moment and shear force are shown in the figure.

When the roof above coal seam breaks, the roof and its upper layer will form the above structure, too. And so on until reach the surface.

#### The cantilever section

In the OA section (-*l* ≤ *x<*0), the shear force and bending moment at *x* can be calculated as *Q*_*x*_ and *M*_*x*_ as shown in Eq (1) and Eq (2).

$$Q_{x}=\left(l+x\right)\cdot q+Q_{A}$$ (1)

$$M_{x}=\frac{1}{2}q{\left(l+x\right)}^{2}+Q_{A}\left(l+x\right)+\frac{h}{2}T$$ (2)

Then the average shear force and tensile stress at *x* can be calculated as *q*_*x*_ and *σ*_*x*_ as shown in Eq (3) and Eq (4).

$$q_{x}=\frac{Q_{x}}{h}=\frac{l+x}{h}\cdot q+\frac{Q_{A}}{h}$$ (3)

$$\sigma_{x}=\frac{6M_{x}}{h^{2}}=3q{\left(\frac{l+x}{h}\right)}^{2}+\frac{6(l+x)Q_{A}}{h^{2}}+\frac{3T}{h}$$ (4)

Their resultant force can be calculated as *f*_*x*_ shown in Eq (5).

$$f_{x}=\sqrt{q_{x}^{2}+\sigma_{x}^{2}}=\frac{1}{h^{2}}\sqrt{{\left[\left(l+x\right)qh+Q_{A}h\right]}^{2}+{\left[3q{\left(l+x\right)}^{2}+6\left(l+x\right)Q_{A}+3hT\right]}^{2}}$$ (5)

The angle between *f*_*x*_ and vertical direction can be calculated as *α*_*x*_ as shown in Eq (6). If the roof break at *x*, the crack initiation angle is equal to *α*_*x*_.

$$\alpha_{x}=\arctan\frac{\sigma_{x}}{q_{x}}=\arctan\frac{3q{\left(l+x\right)}^{2}+6Q_{A}\left(l+x\right)+3hT}{h\left[\left(l+x\right)q+Q_{A}\right]}$$ (6)

#### The semi-infinite elastic foundation beam section

In the OB section (*x*≥0), the coal seam or the lower strata supports the upper strata as an elastic foundation, and the upper strata is a beam with uniformly distributed load, whose force equation is shown in Eq (7). Where, *k* is the foundation coefficient (the pressure required to cause the foundation to produce unit subsidence), *y* is the subsidence of the beam.

$$EIy^{\left(4\right)}=q-ky$$ (7)

By using the method of initial parameters [23], the deflection, rotation angle, bending moment and shear force at *x* can be calculated as *y*_*x*_, *θ*_*x*_, *M*_*x*_ and *Q*_*x*_ as shown in Eq (8), Eq (9), Eq (10) and Eq (11), respectively.

$$y_{x}=y_{0}\cdot \phi_{1}(\beta x)+\frac{\theta_{0}}{\beta }\phi_{2}(\beta x)-\frac{M_{0}}{EI\beta^{2}}\phi_{3}(\beta x)-\frac{Q_{0}}{EI\beta^{3}}\phi_{4}(\beta x)+\frac{q}{k}[1-\phi_{1}(\beta x)]$$ (8)

$$\theta_{x}=\frac{dy}{dx}=-y_{0}\cdot 4\beta \phi_{4}(\beta x)+\theta_{0}\phi_{1}(\beta x)-\frac{M_{0}}{EI\beta }\phi_{2}(\beta x)-\frac{Q_{0}}{EI\beta^{2}}\phi_{3}(\beta x)+\frac{4\beta q}{k}\phi_{4}(\beta x)$$ (9)

$$M_{x}=-EI\frac{d\theta }{dx}=y_{0}\cdot 4EI\beta^{2}\phi_{3}(\beta x)+\theta_{0}\cdot 4EI\beta \phi_{4}(\beta x)+M_{0}\cdot \phi_{1}(\beta x)+\frac{Q_{0}}{\beta }\phi_{2}(\beta x)-\frac{q}{\beta^{2}}\phi_{3}(\beta x)$$ (10)

$$Q_{x}=\frac{dM}{dx}=y_{0}\cdot 4EI\beta^{3}\phi_{2}(\beta x)+\theta_{0}\cdot 4EI\beta^{2}\phi_{3}(\beta x)-4\beta M_{0}\cdot \phi_{4}(\beta x)+Q_{0}\cdot \phi_{1}(\beta x)-\frac{q}{\beta }\phi_{2}(\beta x)$$ (11)

Where,

$$\beta =\sqrt[{4}]{k/(4EI)}$$

$$\phi_{1}(\beta x)=ch(\beta x)\cos(\beta x)$$

$$\phi_{2}(\beta x)=(ch(\beta x)\sin(\beta x)+sh(\beta x)\cos(\beta x))/2$$

$$\phi_{3}(\beta x)=sh(\beta x)\sin(\beta x)/2$$

$$\phi_{4}(\beta x)=(ch(\beta x)\sin(\beta x)-sh(\beta x)\cos(\beta x))/4$$

The deflection and rotation angle far enough away from the O are both 0. That is, while *x* → ∞, $sh\beta x=ch\beta x=\frac{e^{\beta x}}{2}$, $y_{\infty }=\frac{q}{k},\theta_{\infty }=0$. Plug them into Eq (8) and Eq (9), we can get Eq (12) and Eq (13).

$$y_{0}=\frac{q}{k}-\frac{Q_{0}}{2EI\beta^{3}}-\frac{M_{0}}{2EI\beta^{2}}$$ (12)

$$\theta_{0}=\frac{Q_{0}}{2EI\beta^{2}}+\frac{M_{0}}{EI\beta }$$ (13)

Plug Eq (12) and Eq (13) into Eq (10) and Eq (11), we can get Eq (14) and Eq (15).

$$Q_{x}=-e^{-\beta x}\cdot \left[Q_{0}\cdot \left(\sin\beta x-\cos\beta x\right)+2\beta M_{0}\cdot \sin\beta x\right]$$ (14)

$$M_{x}=\frac{e^{-\beta x}}{\beta }\cdot \left[Q_{0}\cdot \sin\beta x+\beta M_{0}\cdot \left(\sin\beta x+\cos\beta x\right)\right]$$ (15)

Then the average shear force and tensile stress at *x* can be calculated as *q*_*x*_ and *σ*_*x*_ as shown in Eq (16) and Eq (17).

$$q_{x}=\frac{Q_{x}}{h}=\frac{-e^{-\beta x}}{h}\cdot \left[Q_{0}\cdot \left(\sin\beta x-\cos\beta x\right)+2\beta M_{0}\cdot \sin\beta x\right]$$ (16)

$$\sigma_{x}=\frac{6M_{x}}{h^{2}}=\frac{6e^{-\beta x}}{\beta h^{2}}\cdot \left[Q_{0}\cdot \sin\beta x+\beta M_{0}\cdot \left(\sin\beta x+\cos\beta x\right)\right]$$ (17)

And we can get Eq (18) and Eq (19) from Eq (1) and Eq (2).

$$Q_{0}=ql+Q_{A}$$ (18)

$$M_{0}=\frac{1}{2}ql^{2}+Q_{A}l+\frac{hT}{2}$$ (19)

Plug Eq (18) and Eq (19) into Eq (16) and Eq (17), we can get Eq (20) and Eq (21).

$$q_{x}=\frac{Q_{x}}{h}=\frac{-e^{-\beta x}}{h}\cdot \left[\left(ql+Q_{A}\right)\left(\sin\beta x-\cos\beta x\right)+\beta \cdot \sin\beta x\cdot \left(ql^{2}+2Q_{A}l+hT\right)\right]$$ (20)

$$\sigma_{x}=\frac{6M_{x}}{h^{2}}=\frac{3e^{-\beta x}}{\beta h^{2}}\cdot \left[2\left(ql+Q_{A}\right)\sin\beta x+\beta \cdot \left(\sin\beta x+\cos\beta x\right)\cdot \left(ql^{2}+2Q_{A}l+hT\right)\right]$$ (21)

So the resultant force can be calculated as *f*_*x*_ as shown in Eq (22).

$$f_{x}=\sqrt{q_{x}^{2}+\sigma_{x}^{2}}=\frac{e^{-\beta x}}{h}\sqrt{\begin{array}{l} {\left[\left(ql+Q_{A}\right)\left(\sin\beta x-\cos\beta x\right)+\beta \cdot \sin\beta x\cdot \left(ql^{2}+2Q_{A}l+hT\right)\right]}^{2}+\\ \frac{9}{\beta^{2}h^{2}}{\left[2\left(ql+Q_{A}\right)\sin\beta x+\beta \cdot \left(\sin\beta x+\cos\beta x\right)\cdot \left(ql^{2}+2Q_{A}l+hT\right)\right]}^{2} \end{array}}$$ (22)

The angle between *f*_*x*_ and vertical direction can be calculated as *α*_*x*_ as shown in Eq (23).

$$\alpha_{x}=\arctan\frac{\sigma_{x}}{q_{x}}=\arctan\left(\frac{-3}{\beta h}\cdot \frac{2\left(ql+Q_{A}\right)\sin\beta x+\beta \cdot \left(\sin\beta x+\cos\beta x\right)\cdot \left(ql^{2}+2Q_{A}l+hT\right)}{\left(ql+Q_{A}\right)\left(\sin\beta x-\cos\beta x\right)+\beta \cdot \sin\beta x\cdot \left(ql^{2}+2Q_{A}l+hT\right)}\right)$$ (23)

#### The three-hinged arch structure section

**Fig 21:**
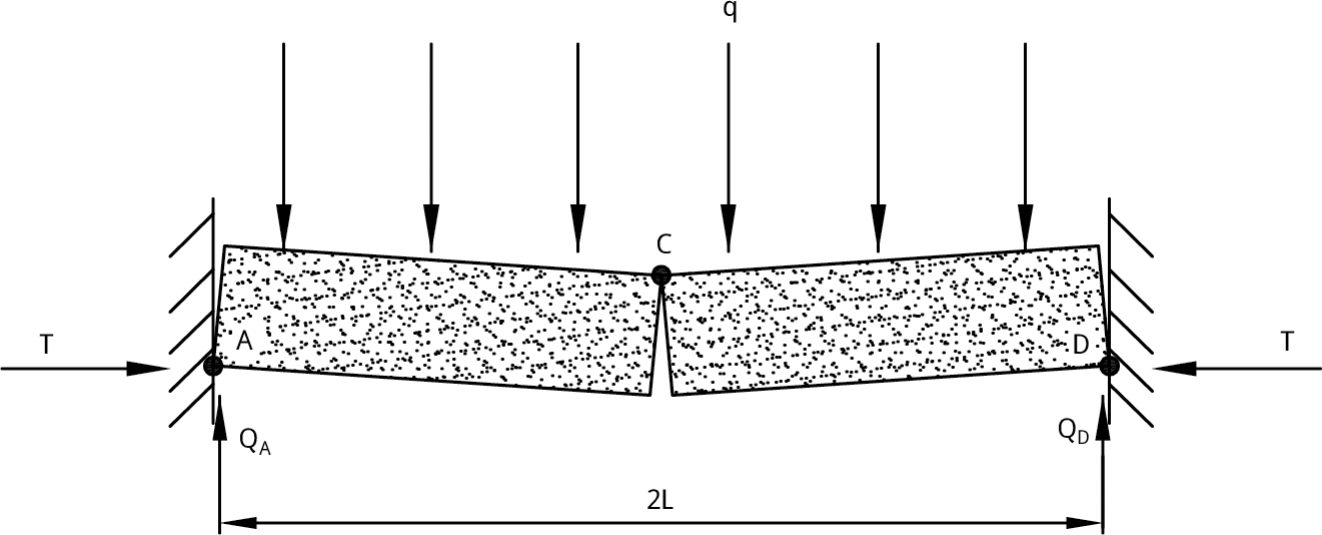
The three-hinged arch structure.

As shown in Fig 21, according to the balance principle of three-hinged arch structure, *T* and *Q*_*A*_ can be calculated by Eq (24) and Eq (25), where *L* is the length of the broken roof.

$$T=\frac{qL^{2}}{2h}$$ (24)

$$Q_{A}=qL$$ (25)

In the case without three-hinged arch structure, *T* and *Q*_*A*_ are all zero.

For easier analysis, the following study all takes the case without three-hinged arch structure as an example.

### Stress distribution in the beam

From Eq (5), we know that, the maximum stress at OA segment is at O, and with the excavation, both *l* and the maximum stress increase gradually. If *l* changes continuously, the O will be the first to reach the tensile strength at OA segment. From Eq (22), we know that, the maximum stress at OB segment is not fixed and must be in front of the O point. So, if *l* changes continuously, somewhere ahead of the O will be the first to reach the tensile strength in the beam.

**Fig 22:**
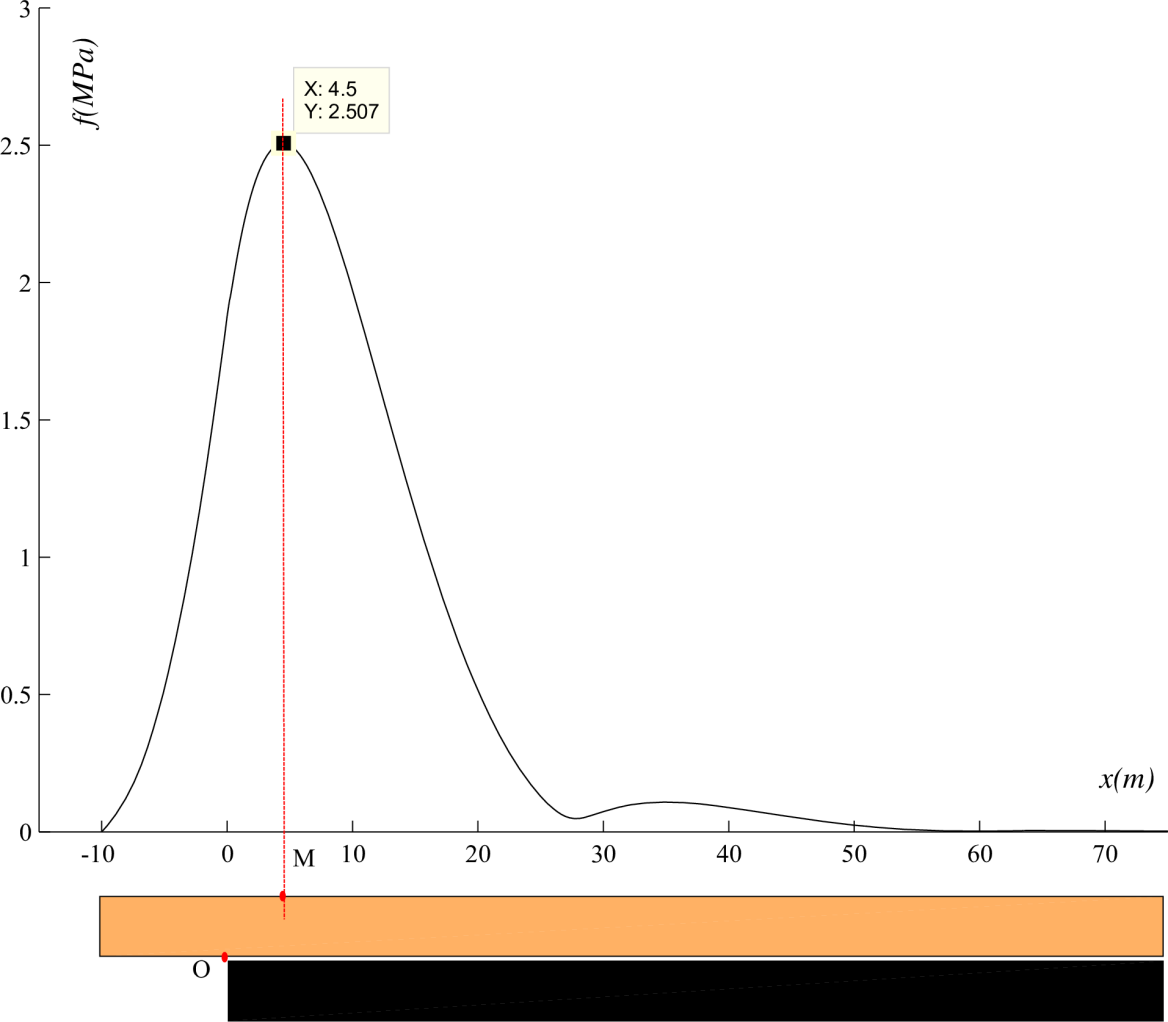
Stress distribution in beam.

In order to observe the stress distribution in the beam more clearly, the example is given by using MATLAB as shown in Fig 22. Where, *q* = 0.46MPa, *k* = 750MN/m^3^, E = 30000MPa, *h* = 8.75m, *l* = 10m. Through the calculation, the maximum stress in the roof is located 4.5m ahead of the coal wall.

### The discontinuity of *l*

The shearer is used to excavate coal in underground coal mining. The cutting depth of drum is generally 0.6m or 0.8m, and the shift step distance of hydraulic support is equal to the cutting depth, so the *l* changes discontinuously and increased by 0.6m or 0.8m. After the roof above coal seam breaks, the hanging length of the strata above the roof will increase abruptly by about ten meters or tens of meters. So the *l* changes discontinuously, which will affect the stress distribution and breakage of the beam.

**Fig 23:**
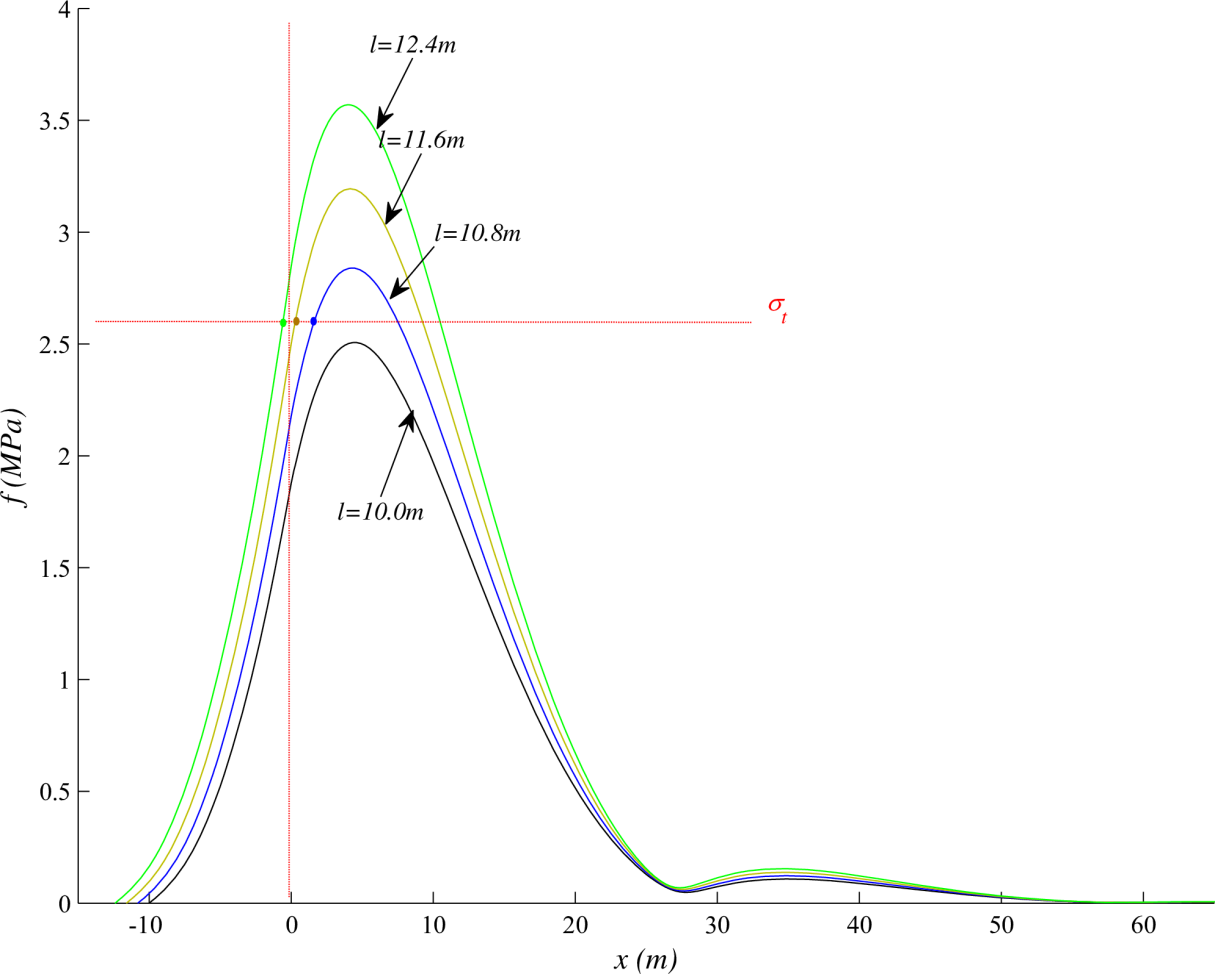
Stress distribution in beam under different *l*.

As shown in Fig 23, we assume the shift step distance is 0.8m. Then we can see that, in the working panel advancing direction, the maximum stress in the roof is always in front of coal panel. If the strata always break at the maximum stress, then the crack initiation point would be always ahead of the coal panel. But the fact is not the case, through the test results and field observation, we know that some crack initiation point is generated in front of the coal panels, and some is behind it. Therefore, we assume that the roof always breaks at the position firstly reaching the tensile strength in the working panel advancing direction, then the fracture position will not exceed the point of the maximum internal force. That is easy to understand, in the gob direction, there are enough space for the roof to bend and break off due to the excavation. But in the working panel advancing direction, the roof is compressed and hardly to deform and break.

As shown in Fig 23, *σ*_*t*_ = 2.6MPa, the maximum internal force in the roof is less than tensile strength and the roof will not break while *l* equals10m. If the shift step distance is 0.8m, *l* would be 10.8m after finishing the next excavation, then the internal force somewhere ahead of the coal panel increases to the tensile strength and the roof breaks. If the shift step distance is 2.4m, *l* would be 12.4m after finishing the next excavation, then the internal force somewhere behind the coal panel increases to the tensile strength and the roof breaks. That is, the relative position of the crack initiation point and the coal panel is not fixed and varies with the different shift step distances. Some crack initiation point is generated in front of the coal panel, and some is in the opposite direction.

### Crack initiation and propagation after roof breaking

**Fig 24:**
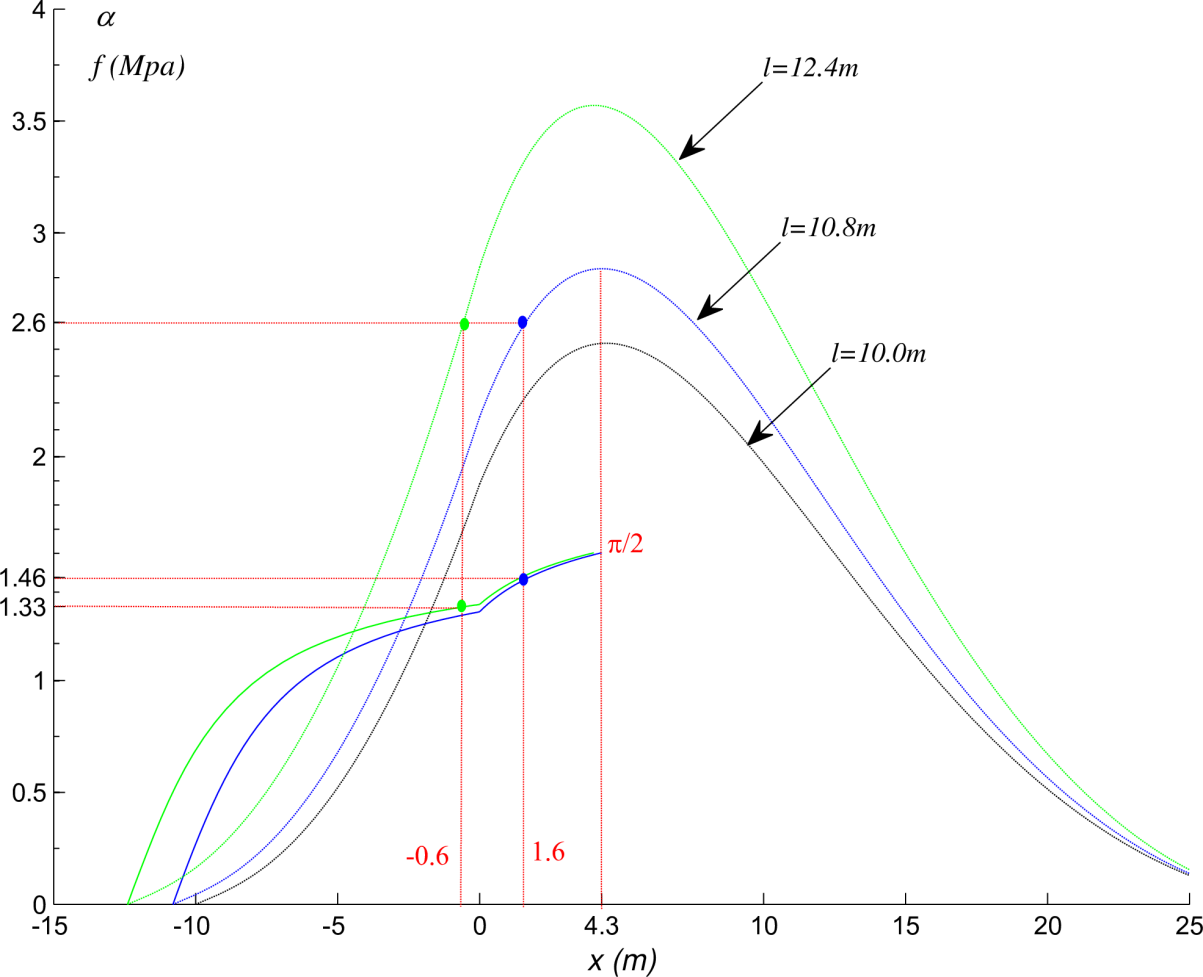
Crack initiation angle under different shift step distance.

Both tensile and shear cracks exit during the damage process [24, 25]. The breakage of the roof is mainly caused by tensile failure, and the direction of crack initiation is always perpendicular to the internal force. Specifically, *l* is determined by the mining state and the excavation step, and the internal force distribution in the beam can be calculated by Eq (5) and Eq (22), and then you can get the position *x* firstly reaching limit tensile strength. By substituting *x* into Eq (6) or Eq (23), we can get the crack initiation angle. Still take the example in Section “Stress distribution in the beam”. If the shift step distance is 0.8m, the roof will break at 1.6m ahead of the coal panel, and the initiation angle is 1.46 radian. If the shift step distance is 2.4m, the roof will break at 0.6m behind the coal panel, and the initiation angle is 1.33 radian (Fig 24).

While advancing to a certain distance, the roof breaks at P and crack will be formed along initiation angle. And the crack propagation direction is affected by overburden-separation. If there is no overburden-separation or less, the direction will be roughly along PO. Otherwise, the stratum will bend and the O will move forward and the fracture angle will be close to the initiation angle and the fault line will be stepped.

### Analysis on the test results

**Fig 25:**
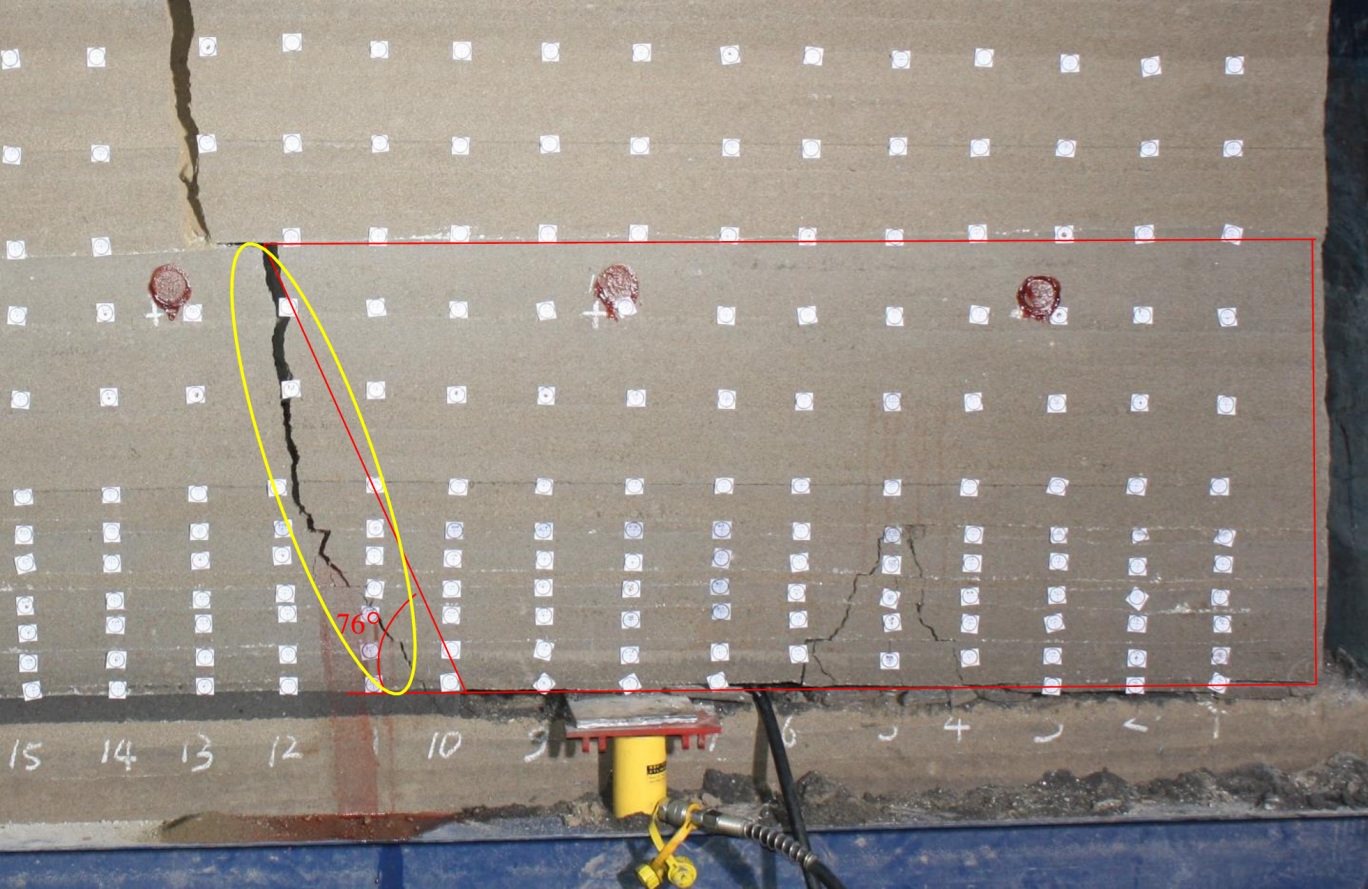
The breakage when excavated to 100cm.

Take the breakage marked in the yellow circle in Fig 25 for example, there is almost no separation of the roof marked in the red circle, which can be calculated as one layer. Then *q* = 0.022MPa, *k* = 7.50MN/m^3^, E = 101MPa, *h* = 0.52m, *σ*_*t*_ = 0.30MPa. When excavated to 100cm, the roof began to fracture in large scale.

**Fig 26:**
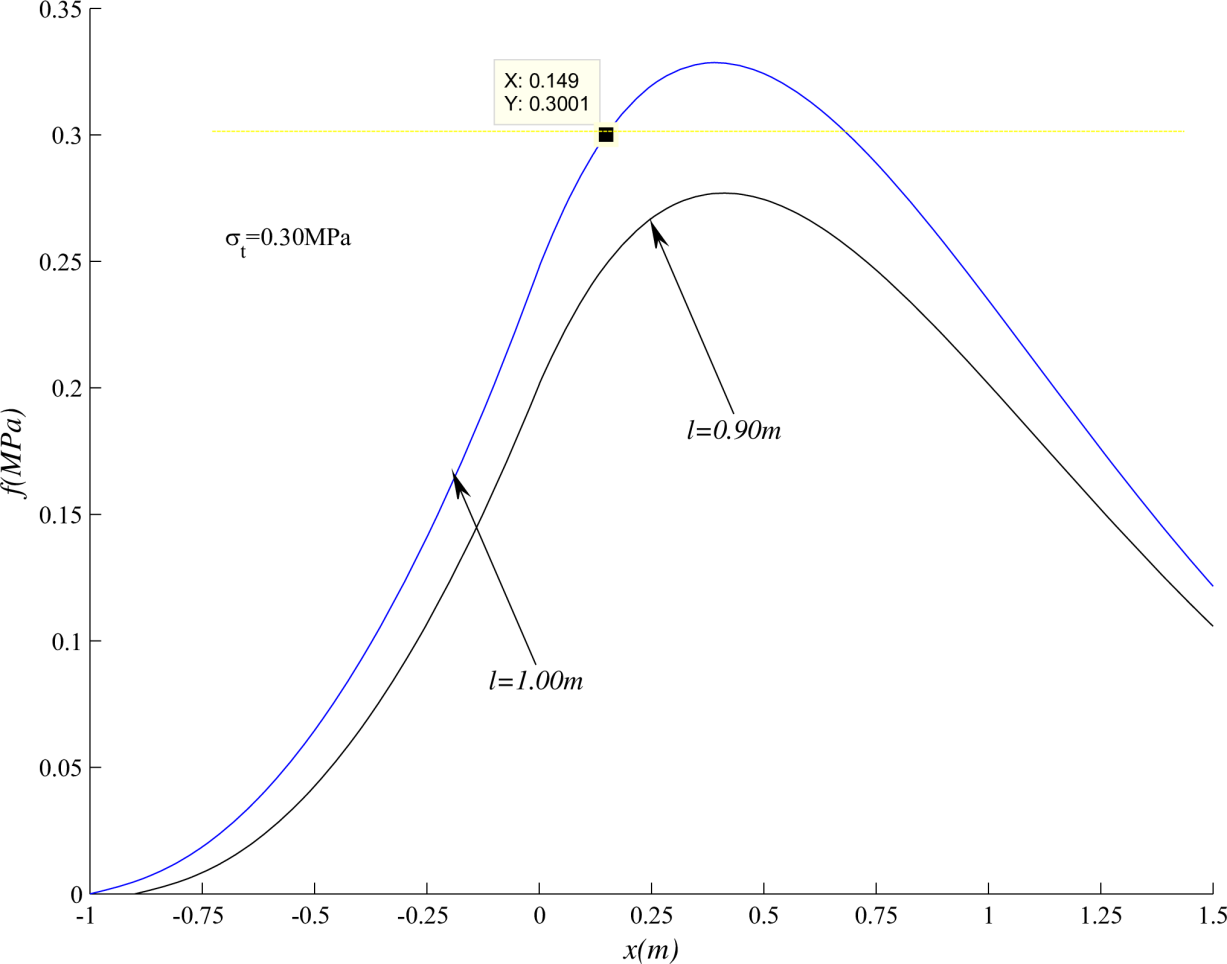
Stress distribution in beam under different *l*.

As shown in Fig 26, through the calculation, the maximum stress in the roof is less than *σ*_*t*_ when excavated to 90cm. After excavated to 100cm, the position where *x* = 0.15m first to reach *σ*_*t*_, so the roof will break at 0.15m ahead of the coal wall (S4 File). Then the crack propagation angle is arc tan(0.52/0.15) = 74°, close to the actual value of 76°.

The simulation test results can be explained as follows by the above mechanical model.

1. With the working face advancing, the *l* gets bigger and bigger, resulting in bigger internal force in the roof. And the roof will break until the maximum internal force reaches the limit tensile strength.
2. The position where the roof’s internal force firstly reaching the tensile strength varies with rock properties and stress conditions of roof, so the range of roof breaking is different. And only when the maximum internal force of all strata reach the limit tensile strength, the breakage can extend to the surface.
3. The hydraulic support offer support load for the roof, so when the coal seam excavates a step forward, the stress distribution of the roof will not change much as long as the supports are not lowered. Thus, overlying strata breakage most likely occurs when the support is lowering.
4. The position where the roof’s internal force firstly reaching the limit tensile strength varies with rock properties and stress conditions of roof and the different shift step distances, and some of which is in front of the coal panel, and some is behind it, so there are two kinds of relative positions between the crack initiation point and the coal panel.
5. The roof breaks at position P where the internal force firstly to reaching the limit tensile strength, and the crack is formed along initiation angle. And then the crack will extend to the supporting point O. Under normal circumstances, in the direction of advancing, if the initiation point P is ahead of the support point O, then the angle between the fault line PO and the horizontal direction will be an obtuse angle. Otherwise, it’s acute.
  Because the maximum internal force is always in the position ahead of the O, if somewhere behind the O is the first place reaching the limit tensile strength and the force is not completely released after breaking, then it’s possible that somewhere ahead of the O still reaches the limit tensile strength and can break again. Because the time is very short, it looks like two different direction fault lines generated at the same time (Fig 12). There are cases as shown in Fig 14, the roof breaks behind the coal panel and gets supported from the hydraulic support during the falling. Then, the O will move backward and dynamic phenomenon occurs, so the roof can break again and generate two different direction fault lines almost at the same time.
6. In fact, the crack propagation is affected by the initiation angle, roof thickness, overburden-separation degree and the position of the O. If there is no overburden-separation or less, the roofs will break as a composite beam and the propagation direction will be roughly along PO. Otherwise, the stratum will bend and the O will move forward and the fracture angle will be close to the initiation angle and the fault line will be stepped (Fig 12 and Fig 16).

## Conclusions

1. By improving analog simulation material property and mounting the artificial pressure devices, two physical simulation tests were conducted and successfully simulated the result that the fault line was in the direction ahead of the coal panel.
2. The mechanical model of “cantilever beam and elastic foundation beam” was proposed to calculate the stress distribution in the overlying strata and explain the mechanisms of ground fissures generation and propagation successfully.
3. The maximum internal force is always in the position ahead of coal panel, but because there are enough space in gob direction for the roof to bend and break due to the excavation, and the roof in the working panel advancing direction is compressed and hardly to deform and break, so the roof always breaks at the position firstly reaching the limit tensile strength in the working panel advancing direction.
4. Because of the shift step distance and the characteristic of roof breakage, the length of the cantilever beam changes discontinuously, resulting in the position P where the internal force firstly reaching the limit tensile strength is not fixed and there will be two different kinds of relative positions between the crack initiation point and the coal wall.
5. Both tensile and shear cracks exit during the damage process. Only tensile crack was talked in this paper, shear crack should be analyzed in the future research.

## Supporting information

S1 File The uniaxial compression experiment data of simulation material No. I. (XLSX)

S2 File The Brazil splitting experimental data of simulation material No. I. (XLSX)

S3 File The stress data and its figure of the hydraulic support during the caving process. (OPJ)

S4 File The calculation code in Matlab of stress distribution in beam under different *l*. (TXT)
